# Supplementary material for: Global epidemiology of occult hepatitis B virus infections in blood donors, a systematic review and meta-analysis
Source: PLoS One. 2022 Aug 22;17(8):e0272920. doi: 10.1371/journal.pone.0272920 (PMC9394819; doi:10.1371/journal.pone.0272920)
Supplement: S2 Appendix — (PDF) [file pone.0272920.s002.pdf]

Appendix S2: Search strategy in PubMed

**Search**	**Virus**
#1 Condition	Occult Hepatitis b OR Occult Viral hepatitis b OR Occult Hepatitis b Virus OR Occult HBV
#2 Population	Blood Donor* OR Blood Donors OR Donor, Blood OR Donors, Blood OR Blood Donation OR Blood Donations OR Donation, Blood OR Donations, Blood OR Blood transfusion OR Transfusion OR Transfusion Transmissible Infections OR Volunteer donors OR Blood Transfusion, Intrauterine OR Blood Transfusion, Autologous OR Transfusion Reaction OR Platelet Transfusion OR Blood Component Transfusion
#3	#1 AND #2
#4	Limit #3 in English and French
