# Supplementary material for: Global epidemiology of occult hepatitis B virus infections in blood donors, a systematic review and meta-analysis
Source: PLoS One. 2022 Aug 22;17(8):e0272920. doi: 10.1371/journal.pone.0272920 (PMC9394819; doi:10.1371/journal.pone.0272920)
Supplement: S4 Appendix — (PDF) [file pone.0272920.s004.pdf]

Appendix S4. Main reasons of exclusion of eligible studies

N°	Author, Year	Title	Reason of exclusion
1	Abd El Kader Mahmoud, 2013	Detection of occult hepatitis B virus infection among blood donors in Sudan.	No data on OBI prevalence or case fatality rate in blood donors
2	Aftab Ahmed, 2012	Transfusion transmitted infections [TTIS] among blood donors of Sukkur.	No data on OBI prevalence or case fatality rate in blood donors
3	Akintule, 2018	Occult HBV Infection in Nigeria.	No data on the number of HBsAg negative tested
4	Allain, 200	Characterization of Occult Hepatitis B Virus Strains in South African Blood Donors.	Selection of study participants with already OBI result known
5	Allain, 2013	Infectivity of blood products from donors with occult hepatitis B virus infection.	Selection of study participants with already OBI result known
6	Alzahrani, 2019	Prevalence of Hepatitis B Virus (HBV) Among Blood Donors in Eastern Saudi Arabia: Results From a Five-Year Retrospective Study of HBV Seromarkers.	No data on the number of HBsAg negative tested
7	Bartelik, 2011	Frequency of hepatitis B and hepatitis C markers and HIV markers detection in blood donors from the Swietokrzyskie region.	No data on OBI prevalence or case fatality rate in blood donors
8	Beltran, 2011	Absence of occult hepatitis B in Colombian blood donors.	No data on OBI prevalence or case fatality rate in blood donors
9	Ben-Chun, 2019	Application of TMA Technology in Donors' HBV-DNA Detection.	No data on OBI prevalence or case fatality rate in blood donors
10	Brojer, 2011	Decade of viral nucleic acid testing (NAT) in Poland (2000- 2010); Methods and yields.	Full text or abstract not found
11	Brojer, 2006	Characterization of HBV DNA+/HBsAg- blood donors in Poland identified by triplex NAT.	No data on the number of HBsAg negative tested
12	Candoti, 2008	Characterization of occult hepatitis B virus from blood donors carrying genotype A2 or genotype D strains.	Selection of study participants with already OBI result known
13	Candotti, 2012	Occult hepatitis B infection in blood donors from South East Asia: molecular characterisation and potential mechanisms of occurrence.	No data on OBI prevalence or case fatality rate in blood donors
14	Carimo, 2018	First report of occult hepatitis B infection among ART naive HIV seropositive individuals in Maputo, Mozambique.	No data on OBI prevalence or case fatality rate in blood donors
15	Chamni, 2014	Genetic characterization and genotyping of hepatitis B virus (HBV) isolates from donors with an occult HBV infection.	Selection of study participants with already OBI result known
16	Compston, 2009	Prevalence of Persistent and Latent Viruses in Untreated Patients Infected With HIV-1 From Ghana, West Africa.	No data on the number of HBsAg negative tested
17	Darmawan, 2015	Seroepidemiology and Occult Hepatitis B Virus Infection in Young Adults in Banjarmasin, Indonesia.	No data on OBI prevalence or case fatality rate in blood donors
18	Das, 2017	Enhancing blood safety through pioneering nat technology in Eastern India.	Duplicates
19	Datta, 2019	Nucleic acid amplification test: Bridging the gap in blood safety & re-evaluation of blood screening for cryptic transfusion-transmitted infection among Indian donors.	No data on the number of HBsAg negative tested
20	Deng, 2020	Alternative hepatitis B virus DNA confirmatory algorithm identified occult hepatitis B virus infection in Chinese blood donors with non-discriminatory nucleic acid testing.	No data on the number of HBsAg negative tested

21	Diarra, 2018	Occult Hepatitis B Virus Infection and Associated Genotypes among HBsAg-negative Subjects in Burkina Faso.	No data on OBI prevalence or case fatality rate in blood donors
22	Dong, 2014	A pilot study on screening blood donors with individual-donation nucleic acid testing in China.	Prevalence determined with repeat donors
23	Dumaidi, 2014	Prevalence of Occult HBV Among Hemodialysis Patients in Two Districts in the Northern Part of the West Bank, Palestine.	No data on OBI prevalence or case fatality rate in blood donors
24	Durro, 2011	Trends in prevalence of hepatitis B virus infection among Albanian blood donors, 1999-2009.	No data on OBI prevalence or case fatality rate in blood donors
25	El-Zaatari, 2007	Hepatitis B virus DNA in serum of 'anti-HBc only'-positive healthy Lebanese blood donors: significance and possible implications.	No data on OBI prevalence or case fatality rate in blood donors
26	Pandey, 2016	Confirmation and follow up of initial "NAT yields": Prospective study from a tertiary healthcare center in India.	Sample size < or = 10 participants
27	Fang, 2014	Hepatitis B virus genotypes, phylogeny and occult infection in a region with a high incidence of hepatocellular carcinoma in China.	Sample size < or = 10 participants
28	Geraldine, 2006	Evidence of occult hepatitis B virus infection among omani blood donors: a premliminary study.	Sample size < or = 10 participants
29	Gessoni, 2014	Significance of anti-HBc only in blood donors: a serological and virological study after hepatitis B vaccination.	No data on OBI prevalence or case fatality rate in blood donors
30	Gonzales, 2010	Efficacy of hepatitis B virus (HBV) DNA screening and characterization of acute and occult HBV infections among blood donors from Madrid, Spain.	Prevalence determined with repeat donors
31	Gonzales Fraile, 2010	Look-back studies in OBI donors. A comparison of two commercial NAT methods.	Prevalence determined with repeat donors
32	Gonzalez, 2020	Overt and occult hepatitis B among immigrants and native blood donors in Madrid, Spain.	Prevalence determined with repeat donors
33	Gou, 2015	Evaluation of an individual-donation nucleic acid amplification testing algorithm for detecting hepatitis B virus infection in Chinese blood donors.	Selection of study participants with already OBI result known
34	Grabarczyk, 2015	Blood donors screening for blood born viruses in Poland.	Review
35	Harvala, 2021	Hepatitis B infections among blood donors in England between 2009 and 2018: Is an occult hepatitis B infection a risk for blood safety?	Prevalence determined with repeat donors
36	Hern, 2020	Occult hepatitisBinfections and anti-HBc prevalence ataresource-limited blood bank in Mexico.	Prevalence determined with repeat donors
37	Huang, 2010	Occult HBV infection in patients with anti-HBc positive alone.	Article not in English or in French
38	Hwang, 2003	Safety of anti-hepatitis B core antibody-positive donors for living-donor liver transplantation.	No data on OBI prevalence or case fatality rate in blood donors
39	Iram, 2009	Seroprevalence of transfusion transmissible infections [TTIS] in blood donors.	No data on OBI prevalence or case fatality rate in blood donors
40	Kaminski, 2006	Evidence of occult hepatitis B virus infection among Omani blood donors: a preliminary study.	No data on the number of HBsAg negative tested
41	Karimi, 2016	Prevalence of antibody to Hepatitis B core antigen and Hepatitis B virus DNA in HBsAg negative healthy blood donors.	Prevalence determined with repeat donors
42	Katchaki, 1978	Serological evidence of presence of HBsAg undetectable by conventional radioimmunoassay in anti-HBc positive blood donors.	Sample size < or = 10 participants
43	Katsoulidou, 2009	Molecular Characterization of Occult Hepatitis B Cases in Greek Blood Donors.	No data on the number of HBsAg negative tested
44	Khin, 2017	Prevalence of hepatitis b core antibody only positive among hepatitis b surface antigen negative blood donors in Mandalay General Hospital, Myanmar.	No data on OBI prevalence or case fatality rate in blood donors

45	Khorami, 2013	Prevalence of HBc-Ab among HBs-Ag negative healthy blood donors in south of Iran.	No data on OBI prevalence or case fatality rate in blood donors
46	Kiely, 2014	Hepatitis B virus nucleic acid amplification testing of Australian blood donors highlights the complexity of confirming occult hepatitis B virus infection.	Prevalence determined with repeat donors
47	Kopacz, 2009	The results of look back and trace back procedures for HBV DNA positive/hbsag negative donors identified in poland.	No data on the number of HBsAg negative tested
48	Kupski, 2008	Serologic and molecular profile of anti-HBc-positive blood bank donors in an area of low endemicity for HBV.	No data on OBI prevalence or case fatality rate in blood donors
49	Larralde, 2013	Hepatitis B escape mutants in Scottish blood donors.	No data on the number of HBsAg negative tested
50	Viet, 2012	Prevalence of hepatitis B & hepatitis C virus infections in potential blood donors in rural Vietnam.	No data on OBI prevalence or case fatality rate in blood donors
51	Lelie, 2016	Detection of different categories of hepatitis B virus (HBV) infection in a multi-regional study comparing the clinical sensitivity of hepatitis B surface antigen and HBV-DNA testing.	No data on the number of HBsAg negative tested
52	Li, 2008	A pilot study for screening blood donors in Taiwan by nucleic acid amplification technology: detecting occult hepatitis B virus infections and closing the serologic window period for hepatitis C virus.	No data on OBI prevalence or case fatality rate in blood donors
53	Liang, 2010	[A cross-sectional survey of occult hepatitis B virus infection in HIV-infected patients in acquired immune deficiency syndrome area].	Article not in English or in French
54	Lieshout-Krikke, 2016	Rare transmission of hepatitis B virus by Dutch donors with occult infection.	Prevalence determined with repeat donors
55	Lieshout-Krikke, 2014	Surface antigen-negative hepatitis B virus infection in Dutch blood donors.	No data on the number of HBsAg negative tested
56	Lin, 2014	Detection and identification of occult HBV in blood donors in Taiwan using a commercial, multiplex, multi-dye nucleic acid amplification technology screening test.	No data on OBI prevalence or case fatality rate in blood donors
57	Lin, 2016	Serological Patterns and Molecular Characterization of Occult Hepatitis B Virus Infection among Blood Donors.	Prevalence determined with repeat donors
58	Lin, 2016	Prevalence of Occult Hepatitis C Virus Infection among Blood Donors in Jiangsu, China.	No data on OBI prevalence or case fatality rate in blood donors
59	Liu, 2003	Clinical aspects and outcomes of volunteer blood donors testing positive for hepatitis-C virus infection in Taiwan: a prospective study.	No data on OBI prevalence or case fatality rate in blood donors
60	Louisirirotchanakul, 2011	Occult hepatitis B virus infection in Thai blood donors.	Selection of study participants with already OBI result known
61	Makroo, 2015	Evaluation of the Procleix Ultrio Plus ID NAT assay for detection of HIV 1, HBV and HCV in blood donors.	No data on OBI prevalence or case fatality rate in blood donors
62	Makroo, 2012	Hepatitis B core antibody testing in Indian blood donors: A double-edged sword!	No data on OBI prevalence or case fatality rate in blood donors
63	Manzini, 2009	Evidence of acute primary occult hepatitis B virus infection in an Italian repeat blood donor.	Case report
64	Martin, 2012	Correlation of improved hepatitis B surface antigen detection limits with hepatitis B virus DNA nucleic acid test yield in blood donations.	No data on OBI prevalence or case fatality rate in blood donors
65	Meilan, 2012	Comparison the effect of individual donation NAT and minipool of 16 donations NAT.	No data on OBI prevalence or case fatality rate in blood donors

66	Men, 2017	[Blood Test Patterns for Blood Donors after Nucleic Acid Detection in the Blood Center].	No data on OBI prevalence or case fatality rate in blood donors
67	Mosley, 1995	Donor screening for antibody to hepatitis B core antigen and hepatitis B virus infection in transfusion recipients.	No data on OBI prevalence or case fatality rate in blood donors
68	Motayo, 2015	Seroprevalence of transfusion transmissible infections (TTI), in first time blood donors in Abeokuta, Nigeria.	No data on OBI prevalence or case fatality rate in blood donors
69	Nishiya, 2021	Occult and active hepatitis B virus detection in donated blood in Sao Paulo, Brazil.	Prevalence determined with repeat donors
70	Nna, 2014	Occult hepatitis B viral infection among blood donors in South-Eastern Nigeria.	Prevalence determined with repeat donors
71	Ol, 2009	PREVALENCE OF HEPATITIS B AND HEPATITIS C VIRUS INFECTIONS IN POTENTIAL BLOOD DONORS IN RURAL CAMBODIA.	No data on OBI prevalence or case fatality rate in blood donors
72	Panhotra, 2005	Occult hepatitis B virus infection among anti-HBc positive blood donors: Necessitates substitution of screening by HBV NAT.	Case report
73	Phan, 2021	Low Genetic Diversity of Hepatitis B Virus Surface Gene amongst Australian Blood Donors.	No data on OBI prevalence or case fatality rate in blood donors
74	Phikulsod, 2009	One-year experience of nucleic acid technology testing for human immunodeficiency virus Type 1, hepatitis C virus, and hepatitis B virus in Thai blood donations.	No data on the number of HBsAg negative tested
75	Pisaturo, 2020	Prevalence of occult HBV infection in Western countries.	Review
76	Punde, 2011	Ascertaining the prevalence of occult hepatitis B virus infection in voluntary blood donors: a study from Central India.	Comment on an article
77	Ramachandra, 2019	Recent and occult hepatitis B virus infections among blood donors in the United States.	Prevalence determined with repeat donors
78	Reesink, 2008	Occult hepatitis B infection in blood donors.	No data on OBI prevalence or case fatality rate in blood donors
79	Ren, 2011	Hepatitis B virus nucleic acid testing in Chinese blood donors with normal and elevated alanine aminotransferase.	No data on the number of HBsAg negative tested
80	Romanò, 2013	Hepatitis B virus infection among first-time blood donors in Italy: prevalence and correlates between serological patterns and occult infection.	No data on OBI prevalence or case fatality rate in blood donors
81	Samardžija, 2020	The impact of positive anti-hbc marker on permanent deferral of voluntary blood donors in eastern croatia and estimation of occult hepatitis b virus infection rate.	No data on OBI prevalence or case fatality rate in blood donors
82	Satake, 2015	Eradication of transfusion-transmitted HBV infection by NAT and Anti-HBc screening in Japan.	No data on OBI prevalence or case fatality rate in blood donors
83	Satake, 2007	Infectivity of blood components with low hepatitis B virus DNA levels identified in a lookback program.	Prevalence determined with repeat donors
84	Scheiblauer, 2020	Detection of hepatitis B virus infection in German blood donors 2008-2015.	Prevalence determined with repeat donors
85	Seed, 2015	Infectivity of blood components from donors with occult hepatitis B infection - results from an Australian lookback programme.	No data on OBI prevalence or case fatality rate in blood donors
86	Seo, 2015	Occult hepatitis B virus infection and blood transfusion.	Review
87	Spreafico, 2015	Poor efficacy of nucleic acid testing in identifying occult HBV infection and consequences for safety of blood supply in Italy.	No data on the number of HBsAg negative tested
88	Stanic, 2017	Three-Year Experience in NAT Screening of Blood Donors for Transfusion Transmitted Viruses in Croatia.	No data on the number of HBsAg negative tested
89	Stolz, 2010	Efficacy of individual nucleic acid amplification testing in reducing the risk of transfusion-transmitted hepatitis B virus infection in Switzerland, a low-endemic region.	No data on the number of HBsAg negative tested

90	Stolz, 2014	Hepatitis B virus DNA viral load determination in hepatitis B surface antigen-negative Swiss blood donors.	Prevalence determined with repeat donors
91	Su, 2011	The clinical significance of occult hepatitis B transfusion in Taiwan - a look-back study.	No data on the number of HBsAg negative tested
92	Tang, 2018	Incidence of hepatitis B virus infection in young Chinese blood donors born after mandatory implementation of neonatal hepatitis B vaccination nationwide.	No data on the number of HBsAg negative tested
93	Tsoi, 2013	Enhanced detection of hepatitis B virus in Hong Kong blood donors after introduction of a more sensitive transcription-mediated amplification assay.	No data on the number of HBsAg negative tested
94	Van De Laar, 2021	Blood donor screening in the Netherlands: Universal anti-HBc screening in combination with HBV nucleic acid amplification testing may allow discontinuation of hepatitis B virus antigen testing.	No data on OBI prevalence or case fatality rate in blood donors
95	Vermeulen, 2019	Reassessment of hepatitis B virus window periods for two transcription-mediated amplification assays using screening data of South African blood donors.	No data on the number of HBsAg negative tested
96	Wang, 2020	Novel hepatitis B virus surface antigen mutations associated with occult genotype B hepatitis B virus infection affect HBsAg detection.	Selection of study participants with already OBI result known
97	Wang, 2020	Occurrence of occult hepatitis B virus infection associated with envelope protein mutations according to anti-HBs carriage in blood donors.	Prevalence determined with repeat donors
98	Wang, 2016	Prevalence of hepatitis B surface antigen (HBsAg) in a blood donor population born prior to and after implementation of universal HBV vaccination in Shenzhen, China.	Prevalence determined with repeat donors
99	Xiao, 2013	Comparative evaluation of a triplex nucleic acid test for detection of HBV DNA, HCV RNA, and HIV-1 RNA, with the Procleix Tigris System.	No data on OBI prevalence or case fatality rate in blood donors
100	Xiuwen, 2017	Serological and virological epidemiology characterization of occult hepatitis B virus infection in Jiaxing volunteer blood donors.	Article not in English or in French
101	Yang, 2010	The efficacy of individual-donation and minipool testing to detect low-level hepatitis B virus DNA in Taiwan.	No data on OBI prevalence or case fatality rate in blood donors
102	Ye, 2013	Six-year pilot study on nucleic acid testing for blood donations in China.	No baseline data for longitudinal study
103	Yoshikawa, 2007	Lengths of hepatitis B viremia and antigenemia in blood donors: preliminary evidence of occult (hepatitis B surface antigen-negative) infection in the acute stage.	No data on OBI prevalence or case fatality rate in blood donors
104	Yuen, 2011	Transmissibility of hepatitis B virus (HBV) infection through blood transfusion from blood donors with occult HBV infection.	No data on the number of HBsAg negative tested
105	Zhang, 2019	Occult HBV infection in Chinese blood donors: role of N-glycosylation mutations and amino acid substitutions in S protein transmembrane domains.	No data on OBI prevalence or case fatality rate in blood donors
106	Zhang, 2018	[Screening of HBV and Positive Population Distribution Characteristics of Voluntary Blood Donors in Qingyang].	Article not in English or in French
107	Zheng, 2019	Blood test strategy of blood donors, ALT and HBsAg HCV-Ab correlation study.	Full text or abstract not found
108	Zheng, 2015	High prevalence of anti-hepatitis B core antigen in hepatitis B virus-vaccinated Chinese blood donors suggests insufficient protection but little threat to the blood supply.	No data on OBI prevalence or case fatality rate in blood donors
109	Zhou, 2008	[Evaluation of multiplex nucleic acid testing assays for screening of hepatitis B virus DNA in blood donation process].	Article not in English or in French
