# Supplementary material for: Global epidemiology of occult hepatitis B virus infections in blood donors, a systematic review and meta-analysis
Source: PLoS One. 2022 Aug 22;17(8):e0272920. doi: 10.1371/journal.pone.0272920 (PMC9394819; doi:10.1371/journal.pone.0272920)
Supplement: S5 Appendix — (PDF) [file pone.0272920.s005.pdf]

Appendix S5: Reference list of included studies on global prevalence of occult hepatitis B infection in blood donors

1. Abbasali P, Mansoor S, Aabdollah J, Mohammad Kazemi A, Farzad O, Keivan S. Detection of HBV DNA in HBsAg negative normal blood donors. 2005. p. 172-6.
2. Abbasi S, Makv, i M, Karimi G, Neisi N. The Prevalence of SEN Virus and Occult Hepatitis B (OBI) Virus Infection Among Blood Donors in Ahvaz City. Jundishapur journal of microbiology. 2016;9(7):e37329-e. PubMed PMID: rayyan-698181372.
3. Akinbami A, Badiru M, Uche E, Onyekwere C, Ismail K, Olowoselu O, et al. The Prevalence of Occult Hepatitis B Infection among Blood Donors in Lagos, Nigeria. Nigerian medical journal : journal of the Nigeria Medical Association. 2019;60(1):22-6. PubMed PMID: rayyan-698181378.
4. Akram A, Islam SMR, Munshi SU, Tabassum S. Detection of Hepatitis B Virus DNA among Chronic and potential Occult HBV patients in resource-limited settings by Loop-Mediated Isothermal Amplification assay. Journal of Viral Hepatitis. 2018;25(11):1306-11. PubMed PMID: rayyan-698181380.
5. Alizadeh Z, Milani S, Sharifi Z. Occult hepatitis B virus infection among Iranian blood donors: a preliminary study. Archives of Iranian medicine. 2014;17(2):106-7. PubMed PMID: rayyan-698181386.
6. Alshayea AI, Eid GE, El-Hazmi MM, Alhetheel AF. Prevalence and characterization of occult hepatitis B infection among blood donors in central Saudi Arabia. Saudi Medical Journal. 2016;37(10):1114-9. PubMed PMID: rayyan-698181402.
7. Aluora PO, Muturi MW, Gachara G. Seroprevalence and genotypic characterization of HBV among low risk voluntary blood donors in Nairobi, Kenya. Virology Journal. 2020;17(1):176-. PubMed PMID: rayyan-698181403.
8. Asim M, Ali R, Khan LA, Husain SA, Singla R, Kar P. Significance of anti-HBc screening of blood donors & its association with occult hepatitis B virus infection: Implications for blood transfusion. 2010. PubMed PMID: rayyan-698181426.
9. Athira KP, Vanathy K, Kulkarni R, Dhodapkar R. The prevalence of occult hepatitis B infection among the blood donors in a tertiary care hospital, Puducherry. Indian journal of medical microbiology. 2018;36(3):426-8. PubMed PMID: rayyan-698181429.
10. Berkem R, Karakoc AE. Safer Blood Supply for Transfusion: Which Algorithm Should Be Used to Determine Occult Hepatitis B Infection in Blood Donors? Clinical Laboratory. 2019;65(5):725-31. PubMed PMID: rayyan-698181450.
11. Bhattacharya P, Ch, ra PK, Datta S, Banerjee A, Chakraborty S, et al. Significant increase in HBV, HCV, HIV and syphilis infections among blood donors in West Bengal, Eastern India 2004-2005: Exploratory screening reveals high frequency of occult HBV infection. World Journal of Gastroenterology. 2007;13(27):3730-3. PubMed PMID: rayyan-698181459.
12. Bhatti FA, Ullah Z, Salamat N, Ayub M, Ghani E. Anti-hepatits B core antigen testing, viral markers, and occult hepatitis B virus infection in Pakistani blood donors: implications for transfusion practice. Transfusion. 2007;47(1):74-9. PubMed PMID: rayyan-698181460.
13. Biswas A, Panigrahi R, Ch, ra PK, Banerjee A, Datta S, et al. Characterization of the occult hepatitis B virus variants circulating among the blood donors from eastern India. TheScientificWorldJournal. 2013;2013:212704-. PubMed PMID: rayyan-698181462.
14. Chang-rong C, Quan Y, Sheng-xiang GE, Shan-hai OU, Hong-ying NI, Yong-chang Z, et al. Surveillance for occult HBV infection and HBsAg variants in blood donors. Chinese Journal of Virology. 2009;(6):178-84. PubMed PMID: rayyan-698181494.

15. Chaurasia R, Rout D, Zaman S, Chatterjee K, ey HC, ra, et al. Comparison of Procleix Ultrio Elite and Procleix Ultrio NAT Assays for Screening of Transfusion Transmitted Infections among Blood Donors in India. International journal of microbiology. 2016;2016:2543156-. PubMed PMID: rayyan-698181496.
16. Chevrier M-C, St-Louis M, Perreault J, Caron B, Castilloux C, Laroche J, et al. Detection and characterization of hepatitis B virus of anti-hepatitis B core antigen-reactive blood donors in Quebec with an in-house nucleic acid testing assay. Transfusion. 2007;47(10):1794-802. PubMed PMID: rayyan-698181512.
17. Das SS. Blood safety through molecular techniques: Pioneering nat technology in Eastern India. Vox Sanguinis. 2019;114:146. PubMed PMID: rayyan-698181530.
18. Delfino CM, Giorgio M, Garcia G, Puch SS, Outon E, Mathet VL. Drug-resistant and immune-escape hepatitis B virus mutants, occult hepatitis B infection and coinfections in public hospital patients from Argentina. Virus Genes. 2021;57(4):327-37. PubMed PMID: rayyan-698181536.
19. Doda V, Arora S, Kirtania T. Serological characterization of occult hepatitis B virus infection among blood donors in India. Transfusion and Apheresis Science. 2014;51(2):162-7. PubMed PMID: rayyan-698181546.
20. Dodd RY, Nguyen ML, Krysztof DE, Notari EP, Stramer SL. Blood donor testing for hepatitis B virus in the United States: is there a case for continuation of hepatitis B surface antigen detection? Transfusion. 2018;58(9):2166-70. PubMed PMID: rayyan-698181547.
21. Duseja A, Sharma S, Subramanian PG, Agnihotri SK, Chakraborti A, Chawla Y. Occult hepatitis B virus (HBV) infection in healthy blood donors. Indian journal of pathology & microbiology. 2003;46(4):690-2. PubMed PMID: rayyan-698181553.
22. El-Ghitany EM, Farghaly AG, Hashish MH. Occult hepatitis B virus infection among hepatitis C virus seropositive and seronegative blood donors in Alexandria, Egypt. The Journal of the Egyptian Public Health Association. 2013;88(1):8-13. PubMed PMID: rayyan-698181559.
23. Fernández-Galindo DA, Galván-Ramírez ML, Andrade-Sierra J, González-Espinoza E, Evangelista-Carrillo LA, Mendoza-Cabrera S, et al. Occult hepatitis B in kidney transplants recipients and donors from Western Mexico. Int J Infect Dis. 2020;91:17-21. PubMed PMID: rayyan-698181578.
24. Fopa D, otti D, Tagny CT, Doux C, Mbanya D, Murphy EL, et al. Occult hepatitis B infection among blood donors from Yaounde, Cameroon. Blood transfusion = Trasfusione del sangue. 2019;17(6):403-8. PubMed PMID: rayyan-698181583.
25. García-Montalvo BM, Farfán-Ale JA, Acosta-Viana KY, Puerto-Manzano FI. Hepatitis B virus DNA in blood donors with anti-HBc as a possible indicator of active hepatitis B virus infection in Yucatan, Mexico. Transfus Med. 2005;15(5):371-8. PubMed PMID: rayyan-698181592.
26. García-Montalvo BM, Ventura-Zapata LP. Molecular and serological characterization of occult hepatitis B infection in blood donors from Mexico. Ann Hepatol. 2011;10(2):133-41. PubMed PMID: rayyan-698181593.
27. Guo Z, Fu P, Yin Y, Wang F, Yin Y, Wang J, et al. The characteristics of hepatitis B surface antigen (HBsAg)-negative hepatitis B virus (HBV) infection in Chinese blood donors: a follow-up study of donors tested negative for HBsAg and reactive for simultaneous nucleic acid testing of HBV, hepatitis C virus, and human immunodeficiency virus. Transfusion. 2017;57(3):832-40. PubMed PMID: rayyan-698181618.
28. Gutierrez C, Devesa M, Loureiro CL, Leon G, Lipr, i F, et al. Molecular and serological evaluation of surface antigen negative hepatitis B virus infection in blood donors from Venezuela. Journal of Medical Virology. 2004;73(2):200-7. PubMed PMID: rayyan-698181622.

29. Hassanshahi G, Arababadi MK, Khoramdelazad H, Yaghini N, Zar, i ER. Assessment of CXCL12 (SDF-1α) polymorphisms and its serum level in posttransfusion occult HBV-infected patients in Southeastern Iran. Arch Med Res. 2010;41(5):338-42. PubMed PMID: rayyan-698181632.
30. Huang CH, Yuan Q, Chen PJ, Zhang YL, Chen CR, Zheng QB, et al. Influence of mutations in hepatitis B virus surface protein on viral antigenicity and phenotype in occult HBV strains from blood donors. J Hepatol. 2012;57(4):720-9. PubMed PMID: rayyan-698181648.
31. Hudu SA, Harmal NS, Saeed MI, Alshrari AS, Malik YA, Niazlin MT, et al. Molecular and serological detection of occult hepatitis B virus among healthy hepatitis B surface antigen-negative blood donors in Malaysia. African health sciences. 2016;16(3):677-83. PubMed PMID: rayyan-698181656.
32. Hui Z. Analysis on serological and molecular biological characteristics of occult HBV infection among qualified young blood donors with anti-HBc positive in Suzhou area. International Journal of Laboratory Medicine. 2017;(12):2990-2. PubMed PMID: rayyan-698181661.
33. Isabel M, Fraile G, Del Bianco AIJ, Guerrero EG, Rivas FVP, Peris LB. Occult hepatitis B infection in blood donors: A real problem. Vox Sanguinis. 2009;96:19. PubMed PMID: rayyan-698181671.
34. Ismail AM, Devakumar S, Anantharam R, Fletcher GJ, Subramani T, John GT, et al. Low frequency of occult hepatitis B infection in anti-HBc seropositive blood donors: experience from a tertiary care centre in South India. Blood transfusion = Trasfusione del sangue. 2012;10(2):230-2. PubMed PMID: rayyan-698181672.
35. Jafarzadeh A, M KA, M M, A P. Occult hepatitis B virus infection among blood donors with antibodies to hepatitis B core antigen. 2008. p. 27-32.
36. Jutavijittum P, Andernach IE, Yousukh A, Samountry B, Samountry K, Thammavong T, et al. Occult hepatitis B infections among blood donors in Lao PDR. Vox Sanguinis. 2014;106(1):31-7. PubMed PMID: rayyan-698181685.
37. Kanwal M, Junaid A, Tariq N. Frequency of true hepatitis B virus positivity in blood donors positive for hepatitis B core antibodies: implications for transfusion practice. JPMA The Journal of the Pakistan Medical Association. 2020;70(9):1559-62. PubMed PMID: rayyan-698181691.
38. Karimabad MN, Hassanshahi G, Arababadi MK, Shabani Z, Shamsizadeh A, Rafatpanah H, et al. Decreased Circulating Level in Parallel With Lack of Associated Genetic Variation in CXCL10 (IP-10) in Southeastern Post-Transfusion Occult HBV-Infected Patients. Labmedicine. 2011;42(7):423-6. PubMed PMID: rayyan-698181693.
39. Keechilot CS, Shenoy V, Kumar A, Biswas L, Vijayrajratnam S, Dinesh K, et al. Detection of occult hepatitis B and window period infection among blood donors by individual donation nucleic acid testing in a tertiary care center in South India. Pathogens and Global Health. 2016;110(7):287-91. PubMed PMID: rayyan-698181698.
40. Khamesipour A, Amiri ZM, Kafiabad SA, Saadat F, Mansour-ghanaei F, Esteghamati A-R, et al. Frequency of hepatitis B virus DNA in anti-HBc positive, HBsAg negative blood donors in Rasht, northern Iran. Transfusion and Apheresis Science. 2011;45(2):195-7. PubMed PMID: rayyan-698181701.
41. Kishk R, Nemr N, Elkady A, our M, Aboelmagd M, Ramsis N, et al. Hepatitis B surface gene variants isolated from blood donors with overt and occult HBV infection in north eastern Egypt. Virology Journal. 2015;12:153-. PubMed PMID: rayyan-698181714.
42. Kleinman SH, Kuhns MC, Todd DS, Glynn SA, McNamara A, DiMarco A, et al. Frequency of HBV DNA detection in US blood donors testing positive for the presence of

anti-HBc: implications for transfusion transmission and donor screening. Transfusion. 2003;43(6):696-704. PubMed PMID: rayyan-698181715.
43. Ks M, R A, P A. The prevalence of the hepatitis B core antibody and the occult hepatitis B infection among voluntary blood donors in chennai, India. Journal of clinical and diagnostic research : JCDR. 2012;6(10):1710-2. PubMed PMID: rayyan-698181721.
44. Liao H, Liu Y, Chen J, Ding W, Li X, Xu Z, et al. Characterization of hepatitis B virus (HBV) preS/S gene mutations in blood donors with occult HBV infection in the Baoji area of North China. Transfusion. 2017;57(3):857-66. PubMed PMID: rayyan-698181752.
45. Lie-Yong S, Li-Qiang FU, Fang F, Pei-Fen Z. Genotyping and mutational analysis of occult hepatitis B virus infection in blood donors of Shaoxing. Chinese Journal of Experimental and Clinical Virology. 2011;(6):470-3. PubMed PMID: rayyan-698181757.
46. Lin KT, Chang CL, Tsai MH, Lin KS, Saldanha J, Hung CM. Detection and identification of occult HBV in blood donors in Taiwan using a commercial, multiplex, multi-dye nucleic acid amplification technology screening test. Vox Sanguinis. 2014;106(2):103-10. PubMed PMID: rayyan-698181761.
47. Mabunda N, Zicai AF, Ismael N, Vubil A, Mello F, Blackard JT, et al. Molecular and serological characterization of occult hepatitis B among blood donors in Maputo, Mozambique. Memorias Do Instituto Oswaldo Cruz. 2020;115:6-. PubMed PMID: rayyan-698181783.
48. Magvansuren TD, Namjil E. Results of screening for occult hepatitis B virus infection among blood donors in Mongolia. Vox Sanguinis. 2015;109:64-5. PubMed PMID: rayyan-698181785.
49. Mahgoub S, otti D, El Ekiaby M, Allain JP. Hepatitis B virus (HBV) infection and recombination between HBV genotypes D and E in asymptomatic blood donors from Khartoum, Sudan. J Clin Microbiol. 2011;49(1):298-306. PubMed PMID: rayyan-698181787.
50. Mahmoud AI, Elsherbiny NM, Afifi NA, Ahmed BM, Yasin AS. Occult Hepatitis B Infection Among Blood Donors in Al Azhar University Hospital, Upper Egypt: The Current Status After 25 years of Vaccine Introduction. The Egyptian journal of immunology. 2018;25(1):45-56. PubMed PMID: rayyan-698181788.
51. Manzini P, Girotto M, Borsotti R, Giachino O, Guaschino R, Lanteri M, et al. Italian blood donors with anti-HBc and occult hepatitis B virus infection. Haematologica. 2007;92(12):1664-70. PubMed PMID: rayyan-698181794.
52. Mardian Y, Yano Y, Wasityastuti W, Ratnasari N, Liang Y, Putri WA, et al. Genetic polymorphisms of HLA-DP and isolated anti-HBc are important subsets of occult hepatitis B infection in Indonesian blood donors: a case-control study. Virology Journal. 2017;14:201-. PubMed PMID: rayyan-698181795.
53. Mehmood A, Raja AJ, Rasool S, Haque IU, NabiTayyab GU. Significance of Testing Anti-HBcIgM Antibodies for the Screening of Hepatitis B in the Donor Blood. Journal of Ayub Medical College, Abbottabad : JAMC. 2020;32(4):S618-S20. PubMed PMID: rayyan-698181805.
54. Moresco MN, Virgolino Hde A, de Morais MP, da Motta-Passos I, Gomes-Gouvêa MS, de Assis LM, et al. Occult hepatitis B virus infection among blood donors from the Brazilian Amazon: implications for transfusion policy. Vox Sang. 2014;107(1):19-25. PubMed PMID: rayyan-698181822.
55. Muselmani W, Habbal W, Monem F. Significance of screening antibodies to hepatitis B virus core antigen among Syrian blood donors. Transfusion Medicine. 2013;23(4):265-8. PubMed PMID: rayyan-698181832.
56. Muselmani W, Habbal W, Monem F. Prevalence of "anti-HBc alone" among Syrian blood donors. Journal of Infection in Developing Countries. 2014;8(8):1013-5. PubMed PMID: rayyan-698181833.

57. Olotu AA, Oyelese AO, Salawu L, Audu RA, Okwuraiwe AP, Aboderin AO. Occult Hepatitis B virus infection in previously screened, blood donors in Ile-Ife, Nigeria: implications for blood transfusion and stem cell transplantation. Virology Journal. 2016;13:76-. PubMed PMID: rayyan-698181845.
58. Oluyinka OO, Tong HV, Bui Tien S, Fagbami AH, Adekanle O, Ojurongbe O, et al. Occult Hepatitis B Virus Infection in Nigerian Blood Donors and Hepatitis B Virus Transmission Risks. PloS one. 2015;10(7):e0131912-e. PubMed PMID: rayyan-698181846.
59. Osuji AI, Agbakoba NR, Ifeanyichukwu MO, Ifeoma E, Idris NA. Hepatitis B Virus Infection and Biomarkers Correlates of Liver Injury among Healthy Blood Donors in Nigeria. 2020;32. PubMed PMID: rayyan-698181849.
60. Panigrahi R, Biswas A, Datta S, Banerjee A, Ch, ra PK, et al. Anti-hepatitis B core antigen testing with detection and characterization of occult hepatitis B virus by an in-house nucleic acid testing among blood donors in Behrampur, Ganjam, Orissa in southeastern India: implications for transfusion. Virology Journal. 2010;7:204-. PubMed PMID: rayyan-698181860.
61. Pisano MB, Blanco S, Carrizo H, Ré VE, Gallego S. Hepatitis B virus infection in blood donors in Argentina: prevalence of infection, genotype distribution and frequency of occult HBV infection. Arch Virol. 2016;161(10):2813-7. PubMed PMID: rayyan-698181868.
62. Rios-Ocampo WA, Cortes-Mancera F, Olarte JC, Soto A, Navas MC. Occult hepatitis B virus infection among blood donors in Colombia. Virol J. 2014;11:206. PubMed PMID: rayyan-698181912.
63. Said ZN, Sayed MH, Salama I, Aboel-Magd EK, Mahmoud MH, Setouhy ME, et al. Occult hepatitis B virus infection among Egyptian blood donors. World J Hepatol. 2013;5(2):64-73. PubMed PMID: rayyan-698181927.
64. Seo DH, Whang DH, Song EY, Kim HS, Park Q. Prevalence of antibodies to hepatitis B core antigen and occult hepatitis B virus infections in Korean blood donors. Transfusion. 2011;51(8):1840-6. PubMed PMID: rayyan-698181965.
65. Shang G, Yan Y, Yang B, Shao C, Wang F, Li Q, et al. Two HBV DNA+/HBsAg- blood donors identified by HBV NAT in Shenzhen, China. Transfusion and Apheresis Science. 2009;41(1):3-7. PubMed PMID: rayyan-698181971.
66. Sharifi Z. Lack of occult hepatitis B virus infection among blood donors in region with high prevalence of HBV in the east of Iran (sistan-o-baluchistan). Vox Sanguinis. 2013;105:94. PubMed PMID: rayyan-698181972.
67. Sheng ZHU, Lanzhen SHI, Guangcheng FU, Lanjuan C. Serological study of blood donors with occult hepatitis B virus infection. Journal of Chinese Physician. 2013;(12):1530-2. PubMed PMID: rayyan-698181974.
68. Silva CM, Costi C, Costa C, Michelon C, Oravec R, Ramos AB, et al. Low rate of occult hepatitis B virus infection among anti-HBc positive blood donors living in a low prevalence region in Brazil. J Infect. 2005;51(1):24-9. PubMed PMID: rayyan-698181981.
69. Sofian M, Aghakhani A, Izadi N, Banifazl M, Kalantar E, Eslamifar A, et al. Lack of occult hepatitis B virus infection among blood donors with isolated hepatitis B core antibody living in an HBV low prevalence region of Iran. International Journal of Infectious Diseases. 2010;14(4):E308-E10. PubMed PMID: rayyan-698181987.
70. Sosa-Jurado F, Hilda Rosas-Murrieta N, Guzman-Flores B, Perez Zempoaltecalt C, Patricia Sanchez Torres A, Ramirez Rosete L, et al. Prevalence of Serologic Hepatitis B Markers in Blood Donors From Puebla, Mexico: The Association of Relatively High Levels of Anti-Core Antibodies With the Detection of Surface Antigen and Genomic DNA. Hepatitis monthly. 2016;16(6):e36942-e. PubMed PMID: rayyan-698181994.

71. Stramer SL, Zou S, Notari EP, Foster GA, Krysztof DE, Musavi F, et al. Blood donation screening for hepatitis B virus markers in the era of nucleic acid testing: are all tests of value? Transfusion. 2012;52(2):440-6. PubMed PMID: rayyan-698182004.
72. Taira R, Satake M, Momose Sy, Hino S, Suzuki Y, Murokawa H, et al. Residual risk of transfusion-transmitted hepatitis B virus (HBV) infection caused by blood components derived from donors with occult HBV infection in Japan. Transfusion. 2013;53(7):1393-404. PubMed PMID: rayyan-698182020.
73. Thedja MD, Roni M, Harahap AR, Siregar NC, Ie SI, Muljono DH. Occult hepatitis B in blood donors in Indonesia: altered antigenicity of the hepatitis B virus surface protein. Hepatology international. 2010;4(3):608-14. PubMed PMID: rayyan-698182029.
74. Vaezjalali M, Rashidpour S, Rezaee H, Hajibeigi B, Zeidi M, Gachkar L, et al. Hepatitis B viral DNA among HBs antigen negative healthy blood donors. Hepatitis monthly. 2013;13(3):e6590-e. PubMed PMID: rayyan-698182042.
75. Wolff FH, Fuchs SC, Br, ão AB. Absence of occult hepatitis B among blood donors in southern Brazil. Braz J Infect Dis. 2011;15(2):159-62. PubMed PMID: rayyan-698182075.
76. Ye X, Li T, Xu X, Du P, Zeng J, Zhu W, et al. Characterisation and follow-up study of occult hepatitis B virus infection in anti-HBc-positive qualified blood donors in southern China. Blood transfusion = Trasfusione del sangue. 2017;15(1):6-12. PubMed PMID: rayyan-698182095.
77. Yong-Ping JI, Long-You Z, Bin Z, Mei-Lian Z, Jie Z, Quan Y. Investigation and analysis of occult hepatitis B virus infection among voluntary blood donors in Lishui area. Chinese Journal of Experimental and Clinical Virology. 2011;(6):457-9. PubMed PMID: rayyan-698182100.
78. Yotsuyanagi H, Yasuda K, Moriya K, Shintani Y, Fujie H, Tsutsumi T, et al. Frequent presence of HBV in the sera of HBsAg-negative, anti-HBc-positive blood donors. Transfusion. 2001;41(9):1093-9. PubMed PMID: rayyan-698182103.
79. Yu DAI, Zhenhua Z, Ling Z, Lei JIN, Binghu SUN, Xu LI. Molecular epidemiological study of occult hepatitis B virus infection in blood donors. Chinese Journal of Infectious Diseases. 2012;(12):235-9. PubMed PMID: rayyan-698182105.
80. Yuan Q, Ou SH, Chen CR, Ge SX, Pei B, Chen QR, et al. Molecular characteristics of occult hepatitis B virus from blood donors in southeast China. J Clin Microbiol. 2010;48(2):357-62. PubMed PMID: rayyan-698182106.
81. Yuen MF, Lee CK, Wong DK, Fung J, Hung I, Hsu A, et al. Prevalence of occult hepatitis B infection in a highly endemic area for chronic hepatitis B: a study of a large blood donor population. Gut. 2010;59(10):1389-93. PubMed PMID: rayyan-698182108.
82. Zheng X, Ye X, Zhang L, Wang W, Shuai L, Wang A, et al. Characterization of occult hepatitis B virus infection from blood donors in China. J Clin Microbiol. 2011;49(5):1730-7. PubMed PMID: rayyan-698182133.
