# Supplementary material for: Global epidemiology of occult hepatitis B virus infections in blood donors, a systematic review and meta-analysis
Source: PLoS One. 2022 Aug 22;17(8):e0272920. doi: 10.1371/journal.pone.0272920 (PMC9394819; doi:10.1371/journal.pone.0272920)
Supplement: S6 Appendix — (PDF) [file pone.0272920.s006.pdf]

Appendix S6. Characteristics of included studies

**Characteristics**	**Overall (87)**	**HBsAg negative and anti-HBc positive (54)**	**HBsAg negative and anti-HBc negative (6)**	**HBsAg negative (27)**
**Year of publication; range**	2001-2021	2001-2021	2004-2018	2003-2020
**Period of inclusion of participants; range**	1991-2019	1991-2019	1995-2016	2003-2018
**Study Design**				
Case control	1 (1.2)			1 (3.7)
Cross-sectional	86 (98.9)	54 (100)	6 (100)	26 (96.3)
**Sampling**				
Non probabilistic	71 (81.6)	45 (83.3)	4 (66.7)	22 (81.5)
Probabilistic	16 (18.4)	9 (16.7)	2 (33.3)	5 (18.5)
**Setting**				
Community-based	1 (1.2)	1 (1.9)		
Hospital-based	86 (98.9)	53 (98.2)	6 (100)	27 (100)
**Number of sites**				
Monocenter	67 (77.0)	43 (79.6)	5 (83.3)	19 (70.4)
Multicenter	20 (23.0)	11 (20.4)	1 (16.7)	8 (29.6)
**Timing of samples collection**				
Prospetively	67 (77.0)	41 (75.9)	6 (100)	20 (74.1)
Retrospectively	20 (23.0)	13 (24.1)		7 (25.9)
**Countries**				
China	15 (17.2)	4 (7.4)		11 (40.7)
India	13 (14.9)	7 (13.0)	1 (16.7)	5 (18.5)
Iran	10 (11.5)	8 (14.8)	1 (16.7)	1 (3.7)
Egypt	5 (5.8)	3 (5.6)	1 (16.7)	1 (3.7)
Mexico	4 (4.6)	3 (5.6)		1 (3.7)
Nigeria	4 (4.6)	1 (1.9)		3 (11.1)
Brazil	3 (3.5)	2 (3.7)	1 (16.7)	
Malaysia	3 (3.5)	2 (3.7)		1 (3.7)
Pakistan	3 (3.5)	3 (5.6)		

**Characteristics**	**Overall (87)**	**HBsAg negative and anti-HBc positive (54)**	**HBsAg negative and anti-HBc negative (6)**	**HBsAg negative (27)**
United States of America	3 (3.5)	3 (5.6)		
Argentina	2 (2.3)	1 (1.9)	1 (16.7)	
Indonesia	2 (2.3)	1 (1.9)		1 (3.7)
Japan	2 (2.3)	2 (3.7)		
Syria	2 (2.3)	2 (3.7)		
Venezuela	2 (2.3)	1 (1.9)	1 (16.7)	
Bangladesh	1 (1.2)	1 (1.9)		
Cameroon	1 (1.2)	1 (1.9)		
Canada	1 (1.2)	1 (1.9)		
Colombia	1 (1.2)	1 (1.9)		
Italy	1 (1.2)	1 (1.9)		
Kenya	1 (1.2)			1 (3.7)
Laos	1 (1.2)	1 (1.9)		
Mongolia	1 (1.2)			1 (3.7)
Mozambique	1 (1.2)			1 (3.7)
Saudi Arabia	1 (1.2)	1 (1.9)		
South Korea	1 (1.2)	1 (1.9)		
Spain	1 (1.2)	1 (1.9)		
Sudan	1 (1.2)	1 (1.9)		
Turkey	1 (1.2)	1 (1.9)		
**WHO Region**				
Western Pacific	23 (26.4)	10 (18.5)		13 (48.2)
Eastern Mediterranean	22 (25.3)	18 (33.3)	2 (33.3)	2 (7.4)
America	16 (18.4)	12 (22.2)	3 (50.0)	1 (3.7)
South-East Asia	16 (18.4)	9 (16.7)	1 (16.7)	6 (22.2)
Africa	7 (8.1)	2 (3.7)		5 (18.5)
Europe	3 (3.5)	3 (5.6)		
**UNSD Region**				
Southern Asia	27 (31.0)	19 (35.2)	2 (33.3)	6 (22.2)

**Characteristics**	**Overall (87)**	**HBsAg negative and anti-HBc positive (54)**	**HBsAg negative and anti-HBc negative (6)**	**HBsAg negative (27)**
Eastern Asia	19 (21.8)	7 (13.0)		12 (44.4)
South America	8 (9.2)	5 (9.3)	3 (50.0)	
Northern Africa	6 (6.9)	4 (7.4)	1 (16.7)	1 (3.7)
Southeastern Asia	6 (6.9)	4 (7.4)		2 (7.4)
Central America	4 (4.6)	3 (5.6)		1 (3.7)
Northern America	4 (4.6)	4 (7.4)		
West Africa	4 (4.6)	1 (1.9)		3 (11.1)
Western Asia	4 (4.6)	4 (7.4)		
Eastern Africa	2 (2.3)			2 (7.4)
Southern Europe	2 (2.3)	2 (3.7)		
Central Africa	1 (1.2)	1 (1.9)		
**Country income level**				
Upper-middle-income economies	43 (49.4)	24 (44.4)	4 (66.7)	15 (55.6)
Lower-middle income economies	30 (34.5)	17 (31.5)	2 (33.3)	11 (40.7)
High-income economies	10 (11.5)	10 (18.5)		
Low-income economies	4 (4.6)	3 (5.6)		1 (3.7)
**Age range**				
Adults	22 (25.3)	14 (25.9)	1 (16.7)	7 (25.9)
All ages	1 (1.2)	1 (1.9)		
Unclear/Not reported	64 (73.6)	39 (72.2)	5 (83.3)	20 (74.1)
**OBI diagnostic method**				
Real-time PCR	59 (67.8)	41 (75.9)	4 (66.7)	14 (51.9)
Classical PCR	27 (31.0)	12 (22.2)	2 (33.3)	13 (48.2)
Loop mediated isothermal amplification (LAMP)	1 (1.2)	1 (1.9)		
**Target detected**				
HBV DNA	87 (100)	54 (100)	6 (100)	27 (100)
**Sample types**				
Blood	87 (100)	54 (100)	6 (100)	27 (100)

Characteristics	Overall (87)	HBsAg negative and anti-HBc positive (54)	HBsAg negative and anti-HBc negative (6)	HBsAg negative (27)
**Risk of bias**				
Low risk of bias	49 (56.3)	31 (57.4)	3 (50.0)	15 (55.6)
Moderate risk of bias	38 (43.7)	23 (42.6)	3 (50.0)	12 (44.4)
