# Supplementary material for: Global epidemiology of occult hepatitis B virus infections in blood donors, a systematic review and meta-analysis
Source: PLoS One. 2022 Aug 22;17(8):e0272920. doi: 10.1371/journal.pone.0272920 (PMC9394819; doi:10.1371/journal.pone.0272920)
Supplement: S7 Appendix — (PDF) [file pone.0272920.s007.pdf]

Appendix S7: Individual characteristics of included studies

Author, Year of publication	Study Design	Sampling	Sampling method	Setting	Number of sites	Timing of samples collection	Countries	WHO Region	UNSD Region	Country income level	Study period	Age range	Population categories	OBI diagnostic method	Target detected	Sample types
Abbasali, 2005	Cross-sectional	Non probabilistic	Consecutive sampling	Hospital-based	Monocenter	Prospetively	Iran	Eastern Mediterranean	Southern Asia	Upper-middle-income economies	Unclear/Not reported	Unclear/Not reported	HBsAg Negative & Anti-HBc Positive	Real-time PCR	HBV DNA	Blood
Abbasi, 2016	Cross-sectional	Non probabilistic	Consecutive sampling	Hospital-based	Monocenter	Prospetively	Iran	Eastern Mediterranean	Southern Asia	Upper-middle-income economies	Oct/2013-Nov/2013	Adults	HBsAg Negative & Anti-HBc Negative	Classical PCR	HBV DNA	Blood
Akinbami, 2019	Cross-sectional	Non probabilistic	Consecutive sampling	Hospital-based	Monocenter	Prospetively	Nigeria	Africa	West Africa	Lower-middle income economies	Nov/2016-Jan/2017	Adults	HBsAg Negative	Classical PCR	HBV DNA	Blood
Akram, 2018	Cross-sectional	Non probabilistic	Consecutive sampling	Hospital-based	Monocenter	Prospetively	Bangladesh	South-East Asia	Southern Asia	Lower-middle income economies	May/2015-May/2016	Adults	HBsAg Negative & Anti-HBc Positive	Loop mediated isothermal amplification (LAMP)	HBV DNA	Blood
Alizadeh, 2014	Cross-sectional	Non probabilistic	Consecutive sampling	Hospital-based	Monocenter	Prospetively	Iran	Eastern Mediterranean	Southern Asia	Upper-middle-income economies	May/2008-Mar/2009	Adults	HBsAg Negative	Real-time PCR	HBV DNA	Blood
Alshayea, 2016	Cross-sectional	Non probabilistic	Consecutive sampling	Hospital-based	Monocenter	Prospetively	Saudi Arabia	Eastern Mediterranean	Western Asia	High-income economies	Jan/2011-Jan/2012	Unclear/Not reported	HBsAg Negative & Anti-HBc Positive	Real-time PCR	HBV DNA	Blood
Aluora, 2020	Cross-sectional	Non probabilistic	Consecutive sampling	Hospital-based	Monocenter	Prospetively	Kenya	Africa	Eastern Africa	Lower-middle income economies	Unclear/Not reported	Adults	HBsAg Negative	Classical PCR	HBV DNA	Blood
Asim, 2010	Cross-sectional	Non probabilistic	Consecutive sampling	Hospital-based	Monocenter	Prospetively	India	South-East Asia	Southern Asia	Lower-middle income economies	Jan/2005-Nov/2006	Adults	HBsAg Negative & Anti-HBc Positive	Classical PCR	HBV DNA	Blood
Athira, 2018	Cross-sectional	Non probabilistic	Consecutive sampling	Hospital-based	Monocenter	Prospetively	India	South-East Asia	Southern Asia	Lower-middle income economies	Jun/2015-Sep/2016	Unclear/Not reported	HBsAg Negative & Anti-HBc Negative	Real-time PCR	HBV DNA	Blood
Athira, 2018	Cross-sectional	Non probabilistic	Consecutive sampling	Hospital-based	Monocenter	Prospetively	India	South-East Asia	Southern Asia	Lower-middle income economies	Jun/2015-Sep/2016	Unclear/Not reported	HBsAg Negative & Anti-HBc Positive	Real-time PCR	HBV DNA	Blood
Berkem, 2019	Cross-sectional	Non probabilistic	Consecutive sampling	Hospital-based	Monocenter	Prospetively	Turkey	Europe	Western Asia	Upper-middle-income economies	Unclear/Not reported	Unclear/Not reported	HBsAg Negative & Anti-HBc Positive	Real-time PCR	HBV DNA	Blood
Bhattacharya, 2007	Cross-sectional	Non probabilistic	Consecutive sampling	Hospital-based	Monocenter	Prospetively	India	South-East Asia	Southern Asia	Lower-middle income economies	Jan/2004-Dec/2005,	Unclear/Not reported	HBsAg Negative & Anti-HBc Positive	Real-time PCR	HBV DNA	Blood
Bhatti, 2007	Cross-sectional	Non probabilistic	Consecutive sampling	Hospital-based	Monocenter	Prospetively	Pakistan	Eastern Mediterranean	Southern Asia	Lower-middle income economies	Jan/2003-Oct/2005	Unclear/Not reported	HBsAg Negative & Anti-HBc Positive	Real-time PCR	HBV DNA	Blood
Biswas, 2013	Cross-sectional	Non probabilistic	Consecutive sampling	Hospital-based	Monocenter	Prospetively	India	South-East Asia	Southern Asia	Lower-middle income economies	Jun/2006-May/2009	Unclear/Not reported	HBsAg Negative & Anti-HBc Positive	Real-time PCR	HBV DNA	Blood
Chang-rong, 2009	Cross-sectional	Non probabilistic	Consecutive sampling	Hospital-based	Monocenter	Prospetively	China	Western Pacific	Eastern Asia	Upper-middle-income economies	Unclear/Not reported	Adults	HBsAg Negative	Classical PCR	HBV DNA	Blood
Chaurasia, 2016	Cross-sectional	Non probabilistic	Convenience sampling	Hospital-based	Multicenter	Retrospectively	India	South-East Asia	Southern Asia	Lower-middle income economies	Jan/2014-Oct/2014	Unclear/Not reported	HBsAg Negative	Real-time PCR	HBV DNA	Blood
Chevrier, 2007	Cross-sectional	Non probabilistic	Consecutive sampling	Hospital-based	Monocenter	Prospetively	Canada	America	Northern America	High-income economies	Oct/2004-Nov/2005	Unclear/Not reported	HBsAg Negative & Anti-HBc Positive	Classical PCR	HBV DNA	Blood
Das, 2019	Cross-sectional	Non probabilistic	Consecutive sampling	Hospital-based	Monocenter	Prospetively	India	South-East Asia	Southern Asia	Lower-middle income economies	Nov/2013-Dec/2018	Unclear/Not reported	HBsAg Negative	Classical PCR	HBV DNA	Blood
Delfino, 2021	Cross-sectional	Non probabilistic	Consecutive sampling	Hospital-based	Monocenter	Retrospectively	Argentina	America	South America	Upper-middle-income economies	2015 -2019	Unclear/Not reported	HBsAg Negative & Anti-HBc Positive	Real-time PCR	HBV DNA	Blood
Doda, 2014	Cross-sectional	Non probabilistic	Consecutive sampling	Hospital-based	Monocenter	Prospetively	India	South-East Asia	Southern Asia	Lower-middle income economies	Jan/2012-Dec/2013	Unclear/Not reported	HBsAg Negative	Real-time PCR	HBV DNA	Blood
Dodd, 2018	Cross-sectional	Non probabilistic	Consecutive sampling	Hospital-based	Multicenter	Prospetively	United States of America	America	Northern America	High-income economies	Jul/2011-Jun/2015	Unclear/Not reported	HBsAg Negative & Anti-HBc Positive	Real-time PCR	HBV DNA	Blood
Duseja, 2003	Cross-sectional	Non probabilistic	Consecutive sampling	Hospital-based	Monocenter	Prospetively	India	South-East Asia	Southern Asia	Lower-middle income economies	Unclear/Not reported	Unclear/Not reported	HBsAg Negative	Classical PCR	HBV DNA	Blood
El-Ghitany, 2013	Case control	Probabilistic	Simple random sampling	Hospital-based	Monocenter	Prospetively	Egypt	Eastern Mediterranean	Northern Africa	Lower-middle income economies	Unclear/Not reported	Unclear/Not reported	HBsAg Negative	Classical PCR	HBV DNA	Blood
Fernández-Galindo, 2020	Cross-sectional	Non probabilistic	Consecutive sampling	Hospital-based	Multicenter	Prospetively	Mexico	America	Central America	Upper-middle-income economies	Unclear/Not reported	Unclear/Not reported	HBsAg Negative	Classical PCR	HBV DNA	Blood
Fopa, 2019	Cross-sectional	Non probabilistic	Consecutive sampling	Hospital-based	Monocenter	Prospetively	Cameroon	Africa	Central Africa	Lower-middle income economies	Aug/2016-Mar/ 2017	Adults	HBsAg Negative & Anti-HBc Positive	Real-time PCR	HBV DNA	Blood
García-Montalvo, 2011	Cross-sectional	Probabilistic	Simple random sampling	Hospital-based	Monocenter	Prospetively	Mexico	America	Central America	Upper-middle-income economies	Jan/2007-Jul/2008	Unclear/Not reported	HBsAg Negative & Anti-HBc Positive	Real-time PCR	HBV DNA	Blood
García-Montalvo, 2005	Cross-sectional	Non probabilistic	Consecutive sampling	Hospital-based	Monocenter	Prospetively	Mexico	America	Central America	Upper-middle-income economies	Jan/1999-Dec/1999	Unclear/Not reported	HBsAg Negative & Anti-HBc Positive	Classical PCR	HBV DNA	Blood
Guo, 2017	Cross-sectional	Non probabilistic	Consecutive sampling	Hospital-based	Monocenter	Retrospectively	China	Western Pacific	Eastern Asia	Upper-middle-income economies	Jun/2011-Oct/2014	Unclear/Not reported	HBsAg Negative	Real-time PCR	HBV DNA	Blood
Gutierrez, 2004	Cross-sectional	Non probabilistic	Consecutive sampling	Hospital-based	Monocenter	Prospetively	Venezuela	America	South America	Upper-middle-income economies	Unclear/Not reported	Unclear/Not reported	HBsAg Negative & Anti-HBc Negative	Classical PCR	HBV DNA	Blood
Gutierrez, 2004	Cross-sectional	Non probabilistic	Consecutive sampling	Hospital-based	Monocenter	Prospetively	Venezuela	America	South America	Upper-middle-income economies	Unclear/Not reported	Unclear/Not reported	HBsAg Negative & Anti-HBc Positive	Classical PCR	HBV DNA	Blood
Hassanshahi, 2010	Cross-sectional	Non probabilistic	Consecutive sampling	Hospital-based	Monocenter	Retrospectively	Iran	Eastern Mediterranean	Southern Asia	Upper-middle-income economies	Unclear/Not reported	Unclear/Not reported	HBsAg Negative & Anti-HBc Positive	Classical PCR	HBV DNA	Blood
Huang, 2012	Cross-sectional	Non probabilistic	Consecutive sampling	Hospital-based	Monocenter	Retrospectively	China	Western Pacific	Eastern Asia	Upper-middle-income economies	2007-2010	Adults	HBsAg Negative	Classical PCR	HBV DNA	Blood
Hudu, 2016	Cross-sectional	Probabilistic	Simple random sampling	Hospital-based	Monocenter	Retrospectively	Malaysia	Western Pacific	Southeastern Asia	Upper-middle-income economies	Unclear/Not reported	Unclear/Not reported	HBsAg Negative & Anti-HBc Negative	Classical PCR	HBV DNA	Blood
Hudu, 2016	Cross-sectional	Probabilistic	Simple random sampling	Hospital-based	Monocenter	Retrospectively	Malaysia	Western Pacific	Southeastern Asia	Upper-middle-income economies	Unclear/Not reported	Unclear/Not reported	HBsAg Negative	Classical PCR	HBV DNA	Blood
Hudu, 2016	Cross-sectional	Probabilistic	Simple random sampling	Hospital-based	Monocenter	Retrospectively	Malaysia	Western Pacific	Southeastern Asia	Upper-middle-income economies	Unclear/Not reported	Unclear/Not reported	HBsAg Negative & Anti-HBc Positive	Classical PCR	HBV DNA	Blood
Hui, 2017	Cross-sectional	Non probabilistic	Consecutive sampling	Hospital-based	Monocenter	Prospetively	China	Western Pacific	Eastern Asia	Upper-middle-income economies	Oct/2013-Jun/2016	Unclear/Not reported	HBsAg Negative & Anti-HBc Positive	Classical PCR	HBV DNA	Blood
Isabel, 2009	Cross-sectional	Non probabilistic	Consecutive sampling	Hospital-based	Monocenter	Retrospectively	Spain	Europe	Southern Europe	High-income economies	Unclear/Not reported	Unclear/Not reported	HBsAg Negative & Anti-HBc Positive	Classical PCR	HBV DNA	Blood
Ismail, 2012	Cross-sectional	Non probabilistic	Consecutive sampling	Hospital-based	Monocenter	Prospetively	India	South-East Asia	Southern Asia	Lower-middle income economies	Oct/2008	All ages	HBsAg Negative & Anti-HBc Positive	Real-time PCR	HBV DNA	Blood
Jafarzadeh, 2008	Cross-sectional	Non probabilistic	Consecutive sampling	Hospital-based	Monocenter	Prospetively	Iran	Eastern Mediterranean	Southern Asia	Upper-middle-income economies	Dec/2004-Dec/2005,	Adults	HBsAg Negative & Anti-HBc Positive	Real-time PCR	HBV DNA	Blood
Jutavijittum, 2014	Cross-sectional	Probabilistic	Simple random sampling	Hospital-based	Monocenter	Prospetively	Laos	Western Pacific	Southeastern Asia	Lower-middle income economies	Mar-Jun/2006	Adults	HBsAg Negative & Anti-HBc Positive	Real-time PCR	HBV DNA	Blood
Kanwal, 2020	Cross-sectional	Non probabilistic	Consecutive sampling	Hospital-based	Monocenter	Retrospectively	Pakistan	Eastern Mediterranean	Southern Asia	Lower-middle income economies	Jun/2014-Dec/2016	Adults	HBsAg Negative & Anti-HBc Positive	Real-time PCR	HBV DNA	Blood
Karimabad, 2011	Cross-sectional	Non probabilistic	Consecutive sampling	Hospital-based	Monocenter	Retrospectively	Iran	Eastern Mediterranean	Southern Asia	Upper-middle-income economies	Unclear/Not reported	Unclear/Not reported	HBsAg Negative & Anti-HBc Positive	Classical PCR	HBV DNA	Blood
Keechilot, 2016	Cross-sectional	Non probabilistic	Consecutive sampling	Hospital-based	Multicenter	Prospetively	India	South-East Asia	Southern Asia	Lower-middle income economies	Mar/2014-Oct/2015	Unclear/Not reported	HBsAg Negative	Real-time PCR	HBV DNA	Blood
Khamesipour, 2011	Cross-sectional	Probabilistic	Simple random sampling	Hospital-based	Monocenter	Retrospectively	Iran	Eastern Mediterranean	Southern Asia	Upper-middle-income economies	Unclear/Not reported	Adults	HBsAg Negative & Anti-HBc Positive	Real-time PCR	HBV DNA	Blood
Kishk, 2015	Cross-sectional	Probabilistic	Simple random sampling	Hospital-based	Monocenter	Retrospectively	Egypt	Eastern Mediterranean	Northern Africa	Lower-middle income economies	Unclear/Not reported	Adults	HBsAg Negative & Anti-HBc Positive	Real-time PCR	HBV DNA	Blood
Kleinman, 2003	Cross-sectional	Non probabilistic	Consecutive sampling	Hospital-based	Multicenter	Retrospectively	United States of America	America	Northern America	High-income economies	1991-1995	Unclear/Not reported	HBsAg Negative & Anti-HBc Positive	Classical PCR	HBV DNA	Blood
Ks, 2012	Cross-sectional	Non probabilistic	Consecutive sampling	Hospital-based	Monocenter	Prospetively	India	South-East Asia	Southern Asia	Lower-middle income economies	2008-2009	Adults	HBsAg Negative & Anti-HBc Positive	Real-time PCR	HBV DNA	Blood
liao, 2017	Cross-sectional	Non probabilistic	Consecutive sampling	Hospital-based	Monocenter	Retrospectively	China	Western Pacific	Eastern Asia	Upper-middle-income economies	Dec/2011-Mar/2015	Unclear/Not reported	HBsAg Negative	Classical PCR	HBV DNA	Blood
Lie-Yong, 2011	Cross-sectional	Non probabilistic	Consecutive sampling	Hospital-based	Monocenter	Prospetively	China	Western Pacific	Eastern Asia	Upper-middle-income economies	Unclear/Not reported	Unclear/Not reported	HBsAg Negative	Classical PCR	HBV DNA	Blood
liu , 2010	Cross-sectional	Probabilistic	Simple random sampling	Hospital-based	Monocenter	Prospetively	China	Western Pacific	Eastern Asia	Upper-middle-income economies	Feb/2007-Apr/2008	Adults	HBsAg Negative	Real-time PCR	HBV DNA	Blood
Mabunda, 2020	Cross-sectional	Non probabilistic	Consecutive sampling	Hospital-based	Monocenter	Prospetively	Mozambique	Africa	Eastern Africa	Low-income economies	Nov/2014-Oct/2015	Unclear/Not reported	HBsAg Negative	Real-time PCR	HBV DNA	Blood
Magvansuren, 2015	Cross-sectional	Non probabilistic	Consecutive sampling	Hospital-based	Monocenter	Prospetively	Mongolia	Western Pacific	Eastern Asia	Lower-middle income economies	2013	Unclear/Not reported	HBsAg Negative	Real-time PCR	HBV DNA	Blood
Mahgoub, 2011	Cross-sectional	Probabilistic	Simple random sampling	Hospital-based	Multicenter	Retrospectively	Sudan	Eastern Mediterranean	Northern Africa	Low-income economies	Feb/2008-Aug/2008	Adults	HBsAg Negative & Anti-HBc Positive	Real-time PCR	HBV DNA	Blood
Mahmoud, 2018	Cross-sectional	Probabilistic	Simple random sampling	Hospital-based	Monocenter	Prospetively	Egypt	Eastern Mediterranean	Northern Africa	Lower-middle income economies	Unclear/Not reported	Unclear/Not reported	HBsAg Negative & Anti-HBc Negative	Real-time PCR	HBV DNA	Blood
Mahmoud, 2018	Cross-sectional	Probabilistic	Simple random sampling	Hospital-based	Monocenter	Prospetively	Egypt	Eastern Mediterranean	Northern Africa	Lower-middle income economies	Unclear/Not reported	Unclear/Not reported	HBsAg Negative & Anti-HBc Positive	Real-time PCR	HBV DNA	Blood
Manzini, 2007	Cross-sectional	Non probabilistic	Consecutive sampling	Hospital-based	Multicenter	Retrospectively	Italy	Europe	Southern Europe	High-income economies	Mar/2005 - Nov/2005	Adults	HBsAg Negative & Anti-HBc Positive	Classical PCR	HBV DNA	Blood
Mardian, 2017	Cross-sectional	Non probabilistic	Consecutive sampling	Hospital-based	Multicenter	Retrospectively	Indonesia	South-East Asia	Southeastern Asia	Upper-middle-income economies	Apr/2013 - Nov/2014	Adults	HBsAg Negative	Real-time PCR	HBV DNA	Blood
Mehmood, 2020	Cross-sectional	Probabilistic	Simple random sampling	Hospital-based	Multicenter	Retrospectively	Pakistan	Eastern Mediterranean	Southern Asia	Lower-middle income economies	Apr/2015 - Jun/2015	Adults	HBsAg Negative & Anti-HBc Positive	Real-time PCR	HBV DNA	Blood
Moresco, 2014	Cross-sectional	Non probabilistic	Consecutive sampling	Hospital-based	Monocenter	Prospetively	Brazil	America	South America	Upper-middle-income economies	Jun/2011-Jun/2012	Unclear/Not reported	HBsAg Negative & Anti-HBc Positive	Real-time PCR	HBV DNA	Blood
Muselmani, 2013	Cross-sectional	Non probabilistic	Consecutive sampling	Hospital-based	Monocenter	Prospetively	Syria	Eastern Mediterranean	Western Asia	Low-income economies	July/2011	Unclear/Not reported	HBsAg Negative & Anti-HBc Positive	Real-time PCR	HBV DNA	Blood
Muselmani, 2014	Cross-sectional	Non probabilistic	Consecutive sampling	Hospital-based	Multicenter	Prospetively	Syria	Eastern Mediterranean	Western Asia	Low-income economies	2011	Unclear/Not reported	HBsAg Negative & Anti-HBc Positive	Real-time PCR	HBV DNA	Blood
Olotu, 2016	Cross-sectional	Non probabilistic	Consecutive sampling	Hospital-based	Monocenter	Prospetively	Nigeria	Africa	West Africa	Lower-middle income economies	Jun/2013-jan/2014	Unclear/Not reported	HBsAg Negative & Anti-HBc Positive	Real-time PCR	HBV DNA	Blood
Oluyinka, 2015	Cross-sectional	Probabilistic	Simple random sampling	Hospital-based	Multicenter	Prospetively	Nigeria	Africa	West Africa	Lower-middle income economies	Unclear/Not reported	Unclear/Not reported	HBsAg Negative	Real-time PCR	HBV DNA	Blood
Osuji, 2020	Cross-sectional	Non probabilistic	Consecutive sampling	Hospital-based	Multicenter	Prospetively	Nigeria	Africa	West Africa	Lower-middle income economies	Unclear/Not reported	Unclear/Not reported	HBsAg Negative	Real-time PCR	HBV DNA	Blood
Panigrahi, 2010	Cross-sectional	Non probabilistic	Consecutive sampling	Community-based	Multicenter	Prospetively	India	South-East Asia	Southern Asia	Lower-middle income economies	Dec/2008-Jul/2009	Unclear/Not reported	HBsAg Negative & Anti-HBc Positive	Real-time PCR	HBV DNA	Blood
Pisano, 2016	Cross-sectional	Non probabilistic	Consecutive sampling	Hospital-based	Monocenter	Prospetively	Argentina	America	South America	Upper-middle-income economies	Jul/2011-Feb/2014	Unclear/Not reported	HBsAg Negative & Anti-HBc Negative	Real-time PCR	HBV DNA	Blood
Rios-Ocampo, 2014	Cross-sectional	Non probabilistic	Convenience sampling	Hospital-based	Monocenter	Prospetively	Colombia	America	South America	Upper-middle-income economies	Feb/2011-Sep/2011	Unclear/Not reported	HBsAg Negative & Anti-HBc Positive	Real-time PCR	HBV DNA	Blood
Said, 2013	Cross-sectional	Non probabilistic	Convenience sampling	Hospital-based	Multicenter	Prospetively	Egypt	Eastern Mediterranean	Northern Africa	Lower-middle income economies	Unclear/Not reported	Adults	HBsAg Negative & Anti-HBc Positive	Real-time PCR	HBV DNA	Blood
Seo, 2011	Cross-sectional	Non probabilistic	Consecutive sampling	Hospital-based	Monocenter	Prospetively	South Korea	Western Pacific	Eastern Asia	High-income economies	Apr/2008-Oct/2008	Unclear/Not reported	HBsAg Negative & Anti-HBc Positive	Real-time PCR	HBV DNA	Blood
Shang, 2009	Cross-sectional	Non probabilistic	Convenience sampling	Hospital-based	Monocenter	Prospetively	China	Western Pacific	Eastern Asia	Upper-middle-income economies	Aug/2006-Dec/2006; Jul/2007-Feb/2008	Unclear/Not reported	HBsAg Negative	Real-time PCR	HBV DNA	Blood
Sharifi, 2013	Cross-sectional	Non probabilistic	Consecutive sampling	Hospital-based	Monocenter	Prospetively	Iran	Eastern Mediterranean	Southern Asia	Upper-middle-income economies	Unclear/Not reported	Unclear/Not reported	HBsAg Negative & Anti-HBc Positive	Real-time PCR	HBV DNA	Blood
Sheng, 2013	Cross-sectional	Non probabilistic	Consecutive sampling	Hospital-based	Multicenter	Prospetively	China	Western Pacific	Eastern Asia	Upper-middle-income economies	Unclear/Not reported	Unclear/Not reported	HBsAg Negative & Anti-HBc Positive	Real-time PCR	HBV DNA	Blood
Silva, 2005	Cross-sectional	Non probabilistic	Consecutive sampling	Hospital-based	Monocenter	Prospetively	Brazil	America	South America	Upper-middle-income economies	2001	Unclear/Not reported	HBsAg Negative & Anti-HBc Positive	Real-time PCR	HBV DNA	Blood
Sofian, 2010	Cross-sectional	Non probabilistic	Consecutive sampling	Hospital-based	Monocenter	Prospetively	Iran	Eastern Mediterranean	Southern Asia	Upper-middle-income economies	2008	Unclear/Not reported	HBsAg Negative & Anti-HBc Positive	Real-time PCR	HBV DNA	Blood
Sosa-Jurado, 2016	Cross-sectional	Non probabilistic	Consecutive sampling	Hospital-based	Multicenter	Prospetively	Mexico	America	Central America	Upper-middle-income economies	2003 - 2009	Unclear/Not reported	HBsAg Negative & Anti-HBc Positive	Real-time PCR	HBV DNA	Blood
Stramer, 2012	Cross-sectional	Non probabilistic	Consecutive sampling	Hospital-based	Monocenter	Prospetively	United States of America	America	Northern America	High-income economies	Jul/2009-Jun/2010	Unclear/Not reported	HBsAg Negative & Anti-HBc Positive	Real-time PCR	HBV DNA	Blood
Taira, 2013	Cross-sectional	Non probabilistic	Consecutive sampling	Hospital-based	Multicenter	Prospetively	Japan	Western Pacific	Eastern Asia	High-income economies	2001-2010	Unclear/Not reported	HBsAg Negative & Anti-HBc Positive	Real-time PCR	HBV DNA	Blood
Thedja, 2010	Cross-sectional	Non probabilistic	Consecutive sampling	Hospital-based	Multicenter	Prospetively	Indonesia	South-East Asia	Southeastern Asia	Upper-middle-income economies	2004–2005	Unclear/Not reported	HBsAg Negative & Anti-HBc Positive	Real-time PCR	HBV DNA	Blood
Vaezjalali, 2013	Cross-sectional	Non probabilistic	Consecutive sampling	Hospital-based	Monocenter	Prospetively	Iran	Eastern Mediterranean	Southern Asia	Upper-middle-income economies	2011	Unclear/Not reported	HBsAg Negative & Anti-HBc Positive	Real-time PCR	HBV DNA	Blood
Wolff, 2011	Cross-sectional	Probabilistic	Simple random sampling	Hospital-based	Multicenter	Prospetively	Brazil	America	South America	Upper-middle-income economies	1995-1997	Unclear/Not reported	HBsAg Negative & Anti-HBc Negative	Real-time PCR	HBV DNA	Blood
Ye, 2017	Cross-sectional	Non probabilistic	Consecutive sampling	Hospital-based	Monocenter	Prospetively	China	Western Pacific	Eastern Asia	Upper-middle-income economies	2014	Adults	HBsAg Negative & Anti-HBc Positive	Real-time PCR	HBV DNA	Blood
Yong-Ping, 2011	Cross-sectional	Non probabilistic	Consecutive sampling	Hospital-based	Monocenter	Prospetively	China	Western Pacific	Eastern Asia	Upper-middle-income economies	Unclear/Not reported	Unclear/Not reported	HBsAg Negative	Classical PCR	HBV DNA	Blood
Yotsuyanagi, 2001	Cross-sectional	Non probabilistic	Consecutive sampling	Hospital-based	Monocenter	Prospetively	Japan	Western Pacific	Eastern Asia	High-income economies	Aug/1997- Mar/1998	Unclear/Not reported	HBsAg Negative & Anti-HBc Positive	Real-time PCR	HBV DNA	Blood
Yu, 2012	Cross-sectional	Non probabilistic	Consecutive sampling	Hospital-based	Monocenter	Prospetively	China	Western Pacific	Eastern Asia	Upper-middle-income economies	Unclear/Not reported	Unclear/Not reported	HBsAg Negative	Classical PCR	HBV DNA	Blood
Yuan, 2010	Cross-sectional	Non probabilistic	Consecutive sampling	Hospital-based	Monocenter	Prospetively	China	Western Pacific	Eastern Asia	Upper-middle-income economies	Jul/2007- 27 Aug/2008	Unclear/Not reported	HBsAg Negative & Anti-HBc Positive	Real-time PCR	HBV DNA	Blood
Yuen, 2010	Cross-sectional	Probabilistic	Simple random sampling	Hospital-based	Multicenter	Retrospectively	China	Western Pacific	Eastern Asia	Upper-middle-income economies	Jun/2005-May/2006	Unclear/Not reported	HBsAg Negative	Real-time PCR	HBV DNA	Blood
Zheng, 2011	Cross-sectional	Non probabilistic	Consecutive sampling	Hospital-based	Multicenter	Prospetively	China	Western Pacific	Eastern Asia	Upper-middle-income economies	Aug/2003-Dec/2009	Unclear/Not reported	HBsAg Negative	Real-time PCR	HBV DNA	Blood
