# Supplementary material for: Global epidemiology of occult hepatitis B virus infections in blood donors, a systematic review and meta-analysis
Source: PLoS One. 2022 Aug 22;17(8):e0272920. doi: 10.1371/journal.pone.0272920 (PMC9394819; doi:10.1371/journal.pone.0272920)
Supplement: S8 Appendix — (PDF) [file pone.0272920.s008.pdf]

Appendix S8: Risk of bias assessment

Author, Year of publication	Was the study's target population a close representation of the national population in relation to OBI prevalence or CFR in blood donors?	Was the sampling frame a true or close representation of the target population?	Was some form of random selection used to select the sample, OR was acensus undertaken?	Were data collected directly from the subjects (as opposed to a proxy)?	Was an acceptable inclusion criteria definition used in the study?	Did the author calculate and respect the expected sample size?	Was the OBI detection assay shown to have reliability and validity?	Was the same mode of data collection used for all subjects?	Was the length of the study period > or = 1 year?	Were the numerator(s) and denominator(s) for the OCI prevalence/CFR appropriate?	Risk of bias	Population categories
Abbasali, 2005	No	Yes	No	Yes	Yes	No	Yes	Yes	Unclear	Yes	Moderate risk of bias	HBsAg Negative & Anti-HBc Positive
Abbasi, 2016	No	Yes	No	Yes	Yes	No	Yes	Yes	No	Yes	Moderate risk of bias	HBsAg Negative & Anti-HBc Negative
Akinbami, 2019	No	Yes	No	Yes	Yes	No	Yes	Yes	No	Yes	Moderate risk of bias	HBsAg Negative
Akram, 2018	No	Yes	No	Yes	Yes	No	Yes	Yes	Yes	Yes	Low risk of bias	HBsAg Negative & Anti-HBc Positive
Alizadeh, 2014	No	Yes	No	Yes	Yes	No	Yes	Yes	No	Yes	Moderate risk of bias	HBsAg Negative
Alshayea, 2016	No	Yes	No	Yes	Yes	No	Yes	Yes	Yes	Yes	Low risk of bias	HBsAg Negative & Anti-HBc Positive
Aluora, 2020	No	Yes	No	Yes	Yes	Yes	Yes	Yes	Unclear	Yes	Low risk of bias	HBsAg Negative
Asim, 2010	No	Yes	No	Yes	Yes	No	Yes	Yes	Yes	Yes	Low risk of bias	HBsAg Negative & Anti-HBc Positive
Athira, 2018	No	Yes	No	Yes	Yes	No	Yes	Yes	Yes	Yes	Low risk of bias	HBsAg Negative & Anti-HBc Negative
Athira, 2018	No	Yes	No	Yes	Yes	No	Yes	Yes	Yes	Yes	Low risk of bias	HBsAg Negative & Anti-HBc Positive
Berkem, 2019	No	Yes	No	Yes	Yes	Unclear	Yes	Yes	Unclear	Yes	Moderate risk of bias	HBsAg Negative & Anti-HBc Positive
Bhattacharya, 2007	No	Yes	No	Yes	Yes	No	Yes	Yes	Yes	Yes	Low risk of bias	HBsAg Negative & Anti-HBc Positive
Bhatti, 2007	No	Yes	No	Yes	Yes	No	Yes	Yes	Yes	Yes	Low risk of bias	HBsAg Negative & Anti-HBc Positive
Biswas, 2013	No	Yes	No	Yes	Yes	No	Yes	Yes	Yes	Yes	Low risk of bias	HBsAg Negative & Anti-HBc Positive
Chang-rong, 2009	No	Yes	No	Yes	Yes	Unclear	Yes	Yes	Unclear	Yes	Moderate risk of bias	HBsAg Negative
Chaurasia, 2016	No	Yes	No	Yes	Yes	No	Yes	Yes	No	Yes	Moderate risk of bias	HBsAg Negative
Chevrier, 2007	No	Yes	No	Yes	Yes	No	Yes	Yes	Yes	Yes	Low risk of bias	HBsAg Negative & Anti-HBc Positive
Das, 2019	No	Yes	No	Yes	Yes	No	Yes	Yes	Yes	Yes	Low risk of bias	HBsAg Negative
Delfino, 2021	No	Yes	No	Yes	Yes	No	Yes	Yes	Yes	Yes	Low risk of bias	HBsAg Negative & Anti-HBc Positive
Doda, 2014	No	Yes	No	Yes	Yes	No	Yes	Yes	Yes	Yes	Low risk of bias	HBsAg Negative
Dodd, 2018	No	Yes	No	Yes	Yes	No	Yes	Yes	Yes	Yes	Low risk of bias	HBsAg Negative & Anti-HBc Positive
Duseja, 2003	No	Yes	Unclear	Yes	Yes	Unclear	Yes	Unclear	Unclear	Yes	Moderate risk of bias	HBsAg Negative
El-Ghitany, 2013	No	Yes	Yes	Yes	Yes	Yes	Yes	Yes	Unclear	Yes	Low risk of bias	HBsAg Negative
Fernández-Galindo, 2020	No	Yes	No	Yes	Yes	No	Yes	Yes	Unclear	Yes	Moderate risk of bias	HBsAg Negative
Fopa, 2019	No	Yes	No	Yes	Yes	No	Yes	Yes	No	Yes	Moderate risk of bias	HBsAg Negative & Anti-HBc Positive
García-Montalvo, 2005	No	Yes	No	Yes	Yes	No	Yes	Yes	Yes	Yes	Low risk of bias	HBsAg Negative & Anti-HBc Positive
García-Montalvo, 2011	No	Yes	Yes	Yes	Yes	No	Yes	Yes	Yes	Yes	Low risk of bias	HBsAg Negative & Anti-HBc Positive
Guo, 2017	No	Yes	No	Yes	Yes	No	Yes	Yes	Yes	Yes	Low risk of bias	HBsAg Negative
Gutierrez, 2004	No	Yes	No	Yes	Yes	No	Yes	Yes	Unclear	Yes	Moderate risk of bias	HBsAg Negative & Anti-HBc Negative
Gutierrez, 2004	No	Yes	No	Yes	Yes	No	Yes	Yes	Unclear	Yes	Moderate risk of bias	HBsAg Negative & Anti-HBc Positive
Hassanshahi, 2010	No	Yes	No	Yes	Yes	No	Yes	Yes	Unclear	Yes	Moderate risk of bias	HBsAg Negative & Anti-HBc Positive
Huang, 2012	No	Yes	No	Yes	Yes	No	Yes	Yes	Yes	Yes	Low risk of bias	HBsAg Negative
Hudu, 2016	No	Yes	Yes	Yes	Yes	No	Yes	Yes	Unclear	Yes	Low risk of bias	HBsAg Negative & Anti-HBc Negative
Hudu, 2016	No	Yes	Yes	Yes	Yes	No	Yes	Yes	Unclear	Yes	Low risk of bias	HBsAg Negative
Hudu, 2016	No	Yes	Yes	Yes	Yes	No	Yes	Yes	Unclear	Yes	Low risk of bias	HBsAg Negative & Anti-HBc Positive
Hui, 2017	No	Yes	No	Yes	Yes	No	Yes	Yes	Yes	Yes	Low risk of bias	HBsAg Negative & Anti-HBc Positive
Isabel, 2009	No	Yes	No	Yes	Yes	No	Yes	Yes	Unclear	Yes	Moderate risk of bias	HBsAg Negative & Anti-HBc Positive
Ismail, 2012	No	Yes	No	Yes	Yes	No	Yes	Yes	No	Yes	Moderate risk of bias	HBsAg Negative & Anti-HBc Positive
Jafarzadeh, 2008	No	Yes	No	Yes	Yes	No	Yes	Yes	Yes	Yes	Low risk of bias	HBsAg Negative & Anti-HBc Positive
Jutavijittum, 2014	No	Yes	Yes	Yes	Yes	No	Yes	Yes	No	Yes	Low risk of bias	HBsAg Negative & Anti-HBc Positive
Kanwal, 2020	No	Yes	No	Yes	Yes	Yes	Yes	Yes	Yes	Yes	Low risk of bias	HBsAg Negative & Anti-HBc Positive
Karimabad, 2011	No	Yes	No	Yes	Yes	No	Yes	Yes	Unclear	Yes	Moderate risk of bias	HBsAg Negative & Anti-HBc Positive
Keechilot, 2016	No	Yes	No	Yes	Yes	No	Yes	Yes	Yes	Yes	Low risk of bias	HBsAg Negative
Khamesipour, 2011	No	Yes	Yes	Yes	Yes	No	Yes	Yes	Unclear	Yes	Low risk of bias	HBsAg Negative & Anti-HBc Positive
Kishk, 2015	No	Yes	Yes	Yes	Yes	No	Yes	Yes	Unclear	Yes	Low risk of bias	HBsAg Negative & Anti-HBc Positive
Kleinman, 2003	No	Yes	No	Yes	Yes	No	Yes	Yes	Yes	Yes	Low risk of bias	HBsAg Negative & Anti-HBc Positive
Ks, 2012	No	Yes	No	Yes	Yes	No	Yes	Yes	Yes	Yes	Low risk of bias	HBsAg Negative & Anti-HBc Positive
liao, 2017	No	Yes	No	Yes	Yes	No	Yes	Yes	Yes	Yes	Low risk of bias	HBsAg Negative
Lie-Yong, 2011	No	Yes	Unclear	Yes	Yes	Unclear	Yes	Yes	Unclear	Yes	Moderate risk of bias	HBsAg Negative
liu , 2010	No	Yes	Yes	Yes	Yes	No	Yes	Yes	Yes	Yes	Low risk of bias	HBsAg Negative
Mabunda, 2020	No	Yes	No	Yes	Yes	No	Yes	Yes	Yes	Yes	Low risk of bias	HBsAg Negative
Magvansuren, 2015	No	Yes	No	Yes	Yes	No	Yes	Yes	Unclear	Yes	Moderate risk of bias	HBsAg Negative
Mahgoub, 2011	No	Yes	Yes	Yes	Yes	No	Yes	Yes	No	Yes	Low risk of bias	HBsAg Negative & Anti-HBc Positive
Mahmoud, 2018	No	Yes	Yes	Yes	Yes	No	Yes	Unclear	Unclear	Yes	Moderate risk of bias	HBsAg Negative & Anti-HBc Negative
Mahmoud, 2018	No	Yes	Yes	Yes	Yes	No	Yes	Unclear	Unclear	Yes	Moderate risk of bias	HBsAg Negative & Anti-HBc Positive
Manzini, 2007	No	Yes	No	Yes	Yes	No	Yes	Yes	No	Yes	Moderate risk of bias	HBsAg Negative & Anti-HBc Positive
Mardian, 2017	No	Yes	No	Yes	Yes	No	Yes	Yes	Yes	Yes	Low risk of bias	HBsAg Negative

Mehmood, 2020	No	Yes	Yes	Yes	Yes	No	Yes	Yes	No	Yes	Low risk of bias	HBsAg Negative & Anti-HBc Positive
Moresco, 2014	No	Yes	No	Yes	Yes	No	Yes	Yes	Yes	Yes	Low risk of bias	HBsAg Negative & Anti-HBc Positive
Muselmani, 2013	No	Yes	No	Yes	Yes	No	Yes	Yes	No	Yes	Moderate risk of bias	HBsAg Negative & Anti-HBc Positive
Muselmani, 2014	No	Yes	No	Yes	Yes	No	Yes	Yes	Unclear	Yes	Moderate risk of bias	HBsAg Negative & Anti-HBc Positive
Olotu, 2016	No	Yes	No	Yes	Yes	No	Yes	Yes	No	Yes	Moderate risk of bias	HBsAg Negative & Anti-HBc Positive
Oluyinka, 2015	No	Yes	Yes	Yes	Yes	No	Yes	Yes	Unclear	Yes	Low risk of bias	HBsAg Negative
Osuji, 2020	No	Yes	No	Yes	Yes	No	Yes	Yes	Unclear	Yes	Moderate risk of bias	HBsAg Negative
Panigrahi, 2010	No	Yes	No	Yes	Yes	No	Yes	Yes	No	Yes	Moderate risk of bias	HBsAg Negative & Anti-HBc Positive
Pisano, 2016	No	Yes	No	Yes	Yes	No	Yes	Yes	Yes	Yes	Low risk of bias	HBsAg Negative & Anti-HBc Negative
Rios-Ocampo, 2014	No	Yes	No	Yes	Yes	No	Yes	Yes	No	Yes	Moderate risk of bias	HBsAg Negative & Anti-HBc Positive
Said, 2013	No	Yes	No	Yes	Yes	No	Yes	Yes	Unclear	Yes	Moderate risk of bias	HBsAg Negative & Anti-HBc Positive
Seo, 2011	No	Yes	No	Yes	Yes	No	Yes	Yes	No	Yes	Moderate risk of bias	HBsAg Negative & Anti-HBc Positive
Shang, 2009	No	Yes	No	Yes	Yes	No	Yes	Yes	No	Yes	Moderate risk of bias	HBsAg Negative
Sharifi, 2013	No	Yes	No	Yes	Yes	Unclear	Yes	Yes	Unclear	Yes	Moderate risk of bias	HBsAg Negative & Anti-HBc Positive
Sheng, 2013	No	Yes	No	Yes	Yes	No	Yes	Yes	Unclear	Yes	Moderate risk of bias	HBsAg Negative & Anti-HBc Positive
Silva, 2005	No	Yes	No	Yes	Yes	No	Yes	Yes	Unclear	Yes	Moderate risk of bias	HBsAg Negative & Anti-HBc Positive
Sofian, 2010	No	Yes	No	Yes	Yes	No	Yes	Yes	Unclear	Yes	Moderate risk of bias	HBsAg Negative & Anti-HBc Positive
Sosa-Jurado, 2016	No	Yes	No	Yes	Yes	No	Yes	Yes	Yes	Yes	Low risk of bias	HBsAg Negative & Anti-HBc Positive
Stramer, 2012	No	Yes	No	Yes	Yes	No	Yes	Yes	Yes	Yes	Low risk of bias	HBsAg Negative & Anti-HBc Positive
Taira, 2013	No	Yes	No	Yes	Yes	No	Yes	Yes	Yes	Yes	Low risk of bias	HBsAg Negative & Anti-HBc Positive
Thedja, 2010	No	Yes	No	Yes	Yes	No	Yes	Yes	Yes	Yes	Low risk of bias	HBsAg Negative & Anti-HBc Positive
Vaezjalali, 2013	No	Yes	No	Yes	Yes	Yes	Yes	Yes	Unclear	Yes	Low risk of bias	HBsAg Negative & Anti-HBc Positive
Wolff, 2011	No	Yes	Yes	Yes	Yes	No	Yes	Yes	Yes	Yes	Low risk of bias	HBsAg Negative & Anti-HBc Negative
Ye, 2017	No	Yes	No	Yes	Yes	No	Yes	Yes	Unclear	Yes	Moderate risk of bias	HBsAg Negative & Anti-HBc Positive
Yong-Ping, 2011	No	Yes	Unclear	Yes	Yes	Unclear	Yes	Yes	Unclear	Yes	Moderate risk of bias	HBsAg Negative
Yotsuyanagi, 2001	No	Yes	No	Yes	Yes	No	Yes	Yes	No	Yes	Moderate risk of bias	HBsAg Negative & Anti-HBc Positive
Yu, 2012	No	Yes	Unclear	Yes	Yes	Unclear	Yes	Yes	Unclear	Yes	Moderate risk of bias	HBsAg Negative
Yuan, 2010	No	Yes	No	Yes	Yes	No	Yes	Yes	Yes	Yes	Low risk of bias	HBsAg Negative & Anti-HBc Positive
Yuen, 2010	No	Yes	Yes	Yes	Yes	No	Yes	Yes	Yes	Yes	Low risk of bias	HBsAg Negative
Zheng, 2011	No	Yes	No	Yes	Yes	No	Yes	Yes	Yes	Yes	Low risk of bias	HBsAg Negative
