# Supplementary figures and images for: Global epidemiology of occult hepatitis B virus infections in blood donors, a systematic review and meta-analysis
Source: PLoS One. 2022 Aug 22;17(8):e0272920. doi: 10.1371/journal.pone.0272920 (PMC9394819; doi:10.1371/journal.pone.0272920)

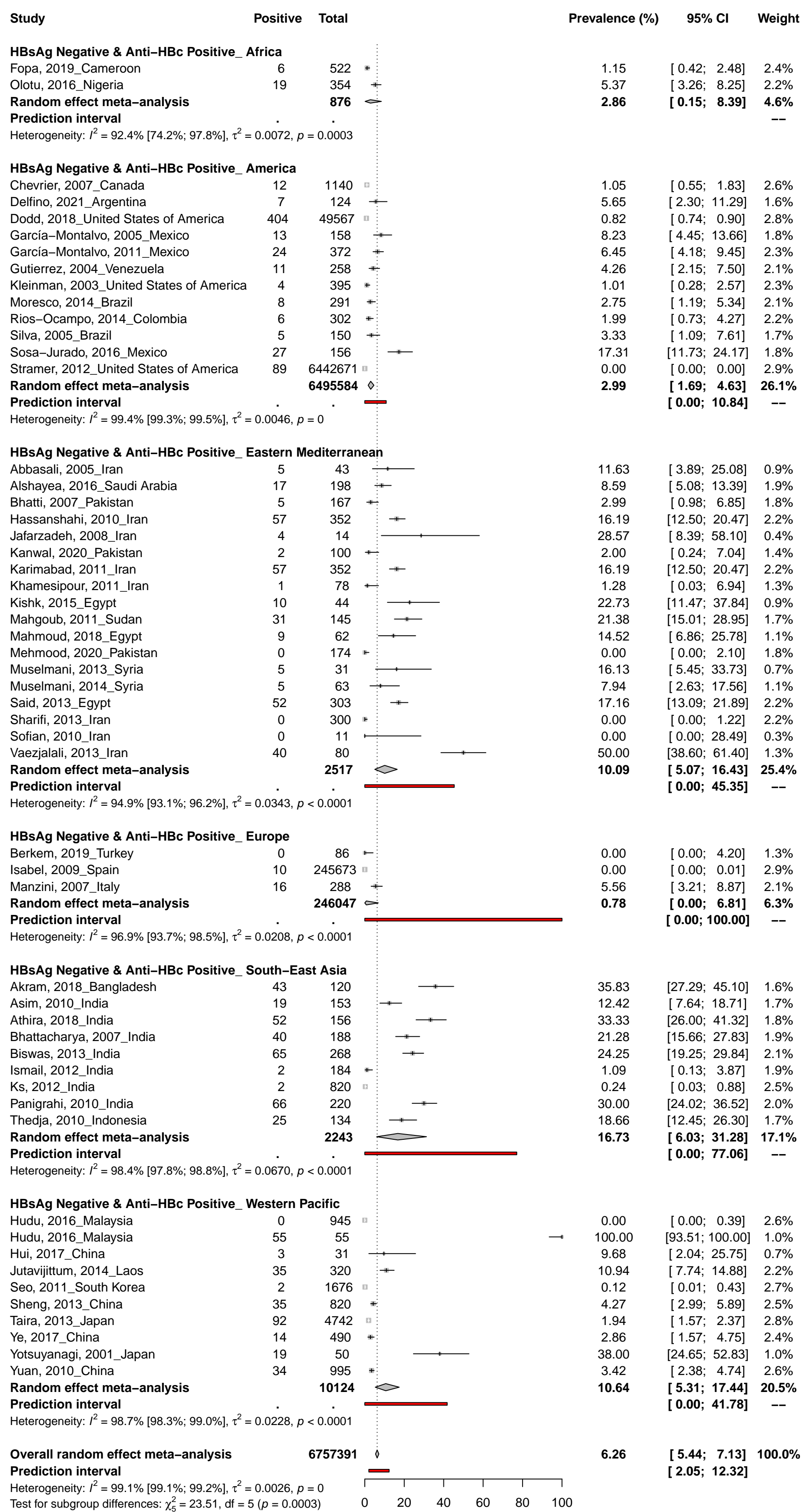

Supplement: S9 Appendix — (PDF) [file pone.0272920.s009.pdf]

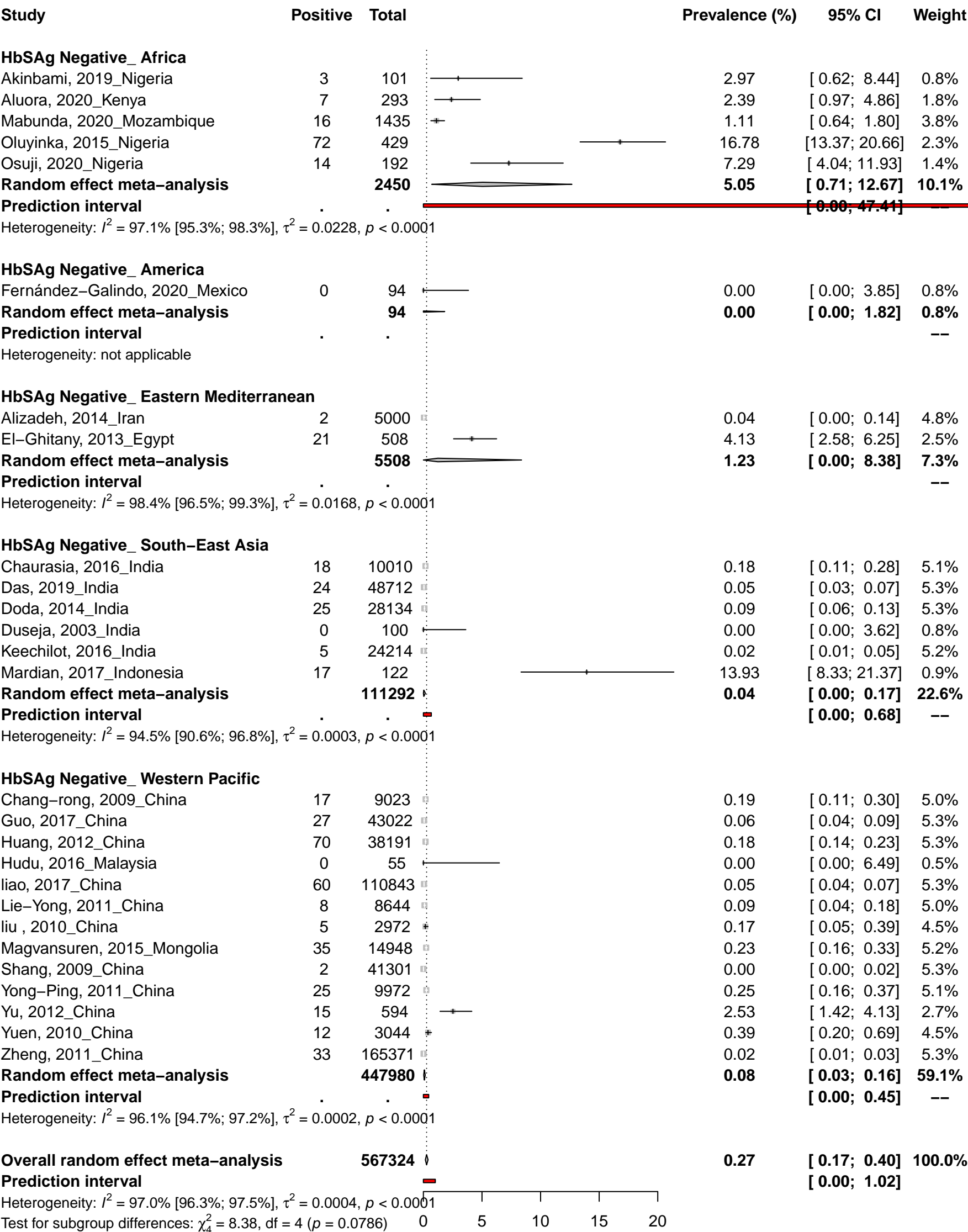

Supplement: S10 Appendix — (PDF) [file pone.0272920.s010.pdf]
