# Supplementary material for: Global epidemiology of occult hepatitis B virus infections in blood donors, a systematic review and meta-analysis
Source: PLoS One. 2022 Aug 22;17(8):e0272920. doi: 10.1371/journal.pone.0272920 (PMC9394819; doi:10.1371/journal.pone.0272920)
Supplement: S11 Appendix — (PDF) [file pone.0272920.s011.pdf]

Appendix S11. Funnel chart for publications of the occult hepatitis B virus prevalence in HBsAg negative & anti-HBc positive blood donors.

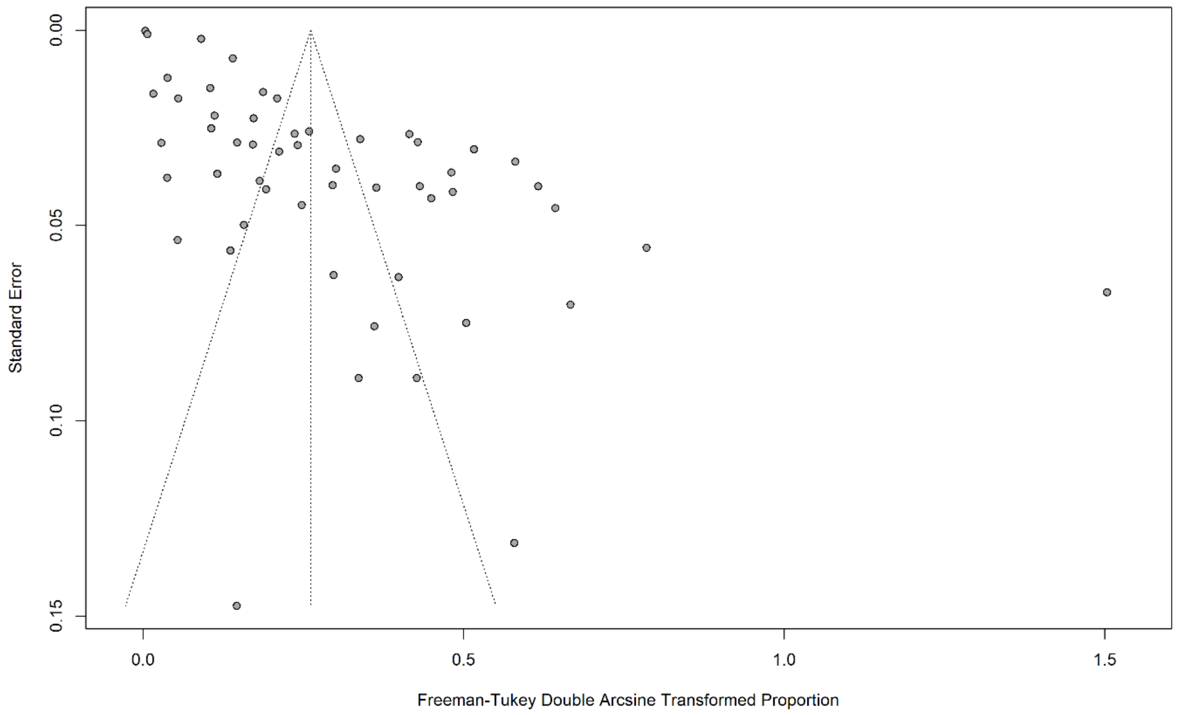

P Egger = < 0.001
