# Supplementary material for: Global epidemiology of occult hepatitis B virus infections in blood donors, a systematic review and meta-analysis
Source: PLoS One. 2022 Aug 22;17(8):e0272920. doi: 10.1371/journal.pone.0272920 (PMC9394819; doi:10.1371/journal.pone.0272920)
Supplement: S14 Appendix — (PDF) [file pone.0272920.s014.pdf]

Appendix S9. Subgroup analyses of global prevalence of occult hepatitis B virus in blood donors.

	Prevalence. % (95%CI)	95% Prediction interval	N Studies	N Participants	H (95%CI)	I² (95%CI)	P heterogeneity	P difference subtypes
**Study Design**								0.028
Case control	4.1 [2.6-6.1]	NA	1	508	NA	NA	1	
Cross-sectional	2.1 [1.8-2.4]	[0.5-4.6]	86	7395817	9.6 [9.2-10]	98.9 [98.8-99]	<0.001	
**Sampling**								0.003
Non probabilistic	1.7 [1.4-2]	[0.3-3.8]	71	7386731	9.6 [9.2-10.1]	98.9 [98.8-99]	<0.001	
Probabilistic	7.3 [3.2-12.8]	[0-38.9]	16	9594	8 [7.2-8.9]	98.5 [98.1-98.7]	<0.001	
**Sampling method**								0.013
Consecutive sampling	1.7 [1.5-2]	[0.4-3.9]	67	7334815	9.7 [9.3-10.1]	98.9 [98.8-99]	<0.001	
Convenience sampling	2 [0.6-4.2]	[0-18.5]	4	51916	9.4 [7.6-11.6]	98.9 [98.3-99.3]	<0.001	
Simple random sampling	7.3 [3.2-12.8]	[0-38.9]	16	9594	8 [7.2-8.9]	98.5 [98.1-98.7]	<0.001	
**Timing of samples collection**								0.669
Prospetively	2.4 [2.1-2.9]	[0.4-5.7]	67	6942313	9.6 [9.2-10]	98.9 [98.8-99]	<0.001	
Retrospectively	2.4 [1.7-3.1]	[0.4-5.6]	20	454012	9.1 [8.4-9.9]	98.8 [98.6-99]	<0.001	
**Countries**								<0.001
Argentina	1.3 [0-12.1]	NA	2	70187	5.4 [3.2-8.8]	96.5 [90.5-98.7]	<0.001	
Bangladesh	35.8 [27.5-44.7]	NA	1	120	NA	NA	1	
Brazil	1.6 [0-4.7]	[0-84.5]	3	594	2.1 [1.2-3.8]	77.4 [26.6-93]	0.012	
Cameroon	1.1 [0.4-2.3]	NA	1	522	NA	NA	1	
Canada	1.1 [0.5-1.7]	NA	1	1140	NA	NA	1	
China	0.3 [0.1-0.4]	[0-1]	15	435313	6.3 [5.5-7.1]	97.5 [96.7-98]	<0.001	
Colombia	2 [0.7-3.9]	NA	1	302	NA	NA	1	
Egypt	9.5 [2.8-19.5]	[0-57.4]	5	1155	4.5 [3.3-6]	95 [90.9-97.2]	<0.001	
India	3.7 [2.5-5.1]	[0.3-10.1]	13	114105	9.2 [8.2-10.2]	98.8 [98.5-99]	<0.001	
Indonesia	16.3 [12-21.2]	NA	2	256	1 NA	1.3 NA	0.314	
Iran	7.2 [0.8-17.7]	[0-58.9]	10	6406	8 [7-9.2]	98.4 [98-98.8]	<0.001	
Italy	5.6 [3.2-8.5]	NA	1	288	NA	NA	1	
Japan	14.7 [0-62.9]	NA	2	4792	7.4 [4.9-11.2]	98.2 [95.9-99.2]	<0.001	
Kenya	2.4 [0.9-4.5]	NA	1	293	NA	NA	1	
Laos	10.9 [7.7-14.6]	NA	1	320	NA	NA	1	
Malaysia	25.1 [0-97]	[0-100]	3	1055	15.2 [12.7-18.3]	99.6 [99.4-99.7]	<0.001	
Mexico	6.5 [1.5-14.2]	[0-57.4]	4	780	3.4 [2.3-5.1]	91.4 [81.2-96.1]	<0.001	

	Prevalence. % (95%CI)	95% Prediction interval	N Studies	N Participants	H (95%CI)	I² (95%CI)	P heterogeneity	P difference subtypes
Mongolia	0.2 [0.2-0.3]	NA	1	14948	NA	NA	1	
Mozambique	1.1 [0.6-1.7]	NA	1	1435	NA	NA	1	
Nigeria	7.7 [2.8-14.6]	[0-51.5]	4	1076	3.5 [2.4-5.2]	91.8 [82.2-96.2]	<0.001	
Pakistan	1.2 [0-4.3]	[0-88]	3	441	2 [1.1-3.6]	74.4 [14.9-92.3]	0.02	
Saudi Arabia	8.6 [5-12.9]	NA	1	198	NA	NA	1	
South Korea	0.1 [0-0.4]	NA	1	1676	NA	NA	1	
Spain	0 [0-0]	NA	1	245673	NA	NA	1	
Sudan	21.4 [15.1-28.5]	NA	1	145	NA	NA	1	
Syria	10.7 [3.9-19.8]	NA	2	94	1.2 NA	29.6 NA	0.233	
Turkey	0 [0-2]	NA	1	86	NA	NA	1	
United States of America	0.4 [0-1.8]	[0-68.2]	3	6492633	27.4 [24.2-30.9]	99.9 [99.8-99.9]	<0.001	
Venezuela	2.2 [0-7.4]	NA	2	292	1.4 NA	49.3 NA	0.16	
**WHO Region**								<0.001
Africa	4.4 [1.3-9]	[0-27.2]	7	3326	5.1 [4.1-6.3]	96.1 [93.9-97.5]	<0.001	
America	1.7 [1.1-2.5]	[0-5.5]	16	6565928	11.4 [10.5-12.4]	99.2 [99.1-99.4]	<0.001	
Eastern Mediterranean	7.4 [3.2-13]	[0-44]	22	8439	6.6 [6-7.3]	97.7 [97.2-98.1]	<0.001	
Europe	0.8 [0-6.8]	[0-100]	3	246047	5.7 [4-8.1]	96.9 [93.7-98.5]	<0.001	
South-East Asia	5.8 [4.3-7.6]	[0.9-14.3]	16	114481	9.5 [8.6-10.4]	98.9 [98.7-99.1]	<0.001	
Western Pacific	0.8 [0.5-1.1]	[0-2.6]	23	458104	8.4 [7.7-9.1]	98.6 [98.3-98.8]	<0.001	
**UNSD Region**								<0.001
Central Africa	1.1 [0.4-2.3]	NA	1	522	NA	NA	1	
Central America	6.5 [1.5-14.2]	[0-57.4]	4	780	3.4 [2.3-5.1]	91.4 [81.2-96.1]	<0.001	
Eastern Africa	1.5 [0.5-3]	NA	2	1728	1.7 [1-3.5]	63.4 [0-91.6]	0.098	
Eastern Asia	0.4 [0.2-0.6]	[0-1.5]	19	456729	7.2 [6.5-8]	98.1 [97.6-98.4]	<0.001	
Northern Africa	11.3 [4.2-21.2]	[0-54.5]	6	1300	4.6 [3.5-5.9]	95.2 [91.9-97.1]	<0.001	
Northern America	0.5 [0-1.8]	[0-13.2]	4	6493773	22.7 [20.2-25.4]	99.8 [99.8-99.8]	<0.001	
South America	1.5 [0-4.6]	[0-18]	8	71375	4.6 [3.7-5.7]	95.2 [92.6-96.9]	<0.001	
Southeastern Asia	19.2 [1.8-47.9]	[0-100]	6	1631	10.8 [9.3-12.6]	99.1 [98.8-99.4]	<0.001	
Southern Asia	4.7 [3.4-6.2]	[0.2-13.4]	27	121072	8.4 [7.8-9.1]	98.6 [98.4-98.8]	<0.001	
Southern Europe	1.4 [0-11.8]	NA	2	245961	8 [5.4-11.8]	98.4 [96.5-99.3]	<0.001	
West Africa	7.7 [2.8-14.6]	[0-51.5]	4	1076	3.5 [2.4-5.2]	91.8 [82.2-96.2]	<0.001	
Western Asia	6 [0.7-14.9]	[0-62.4]	4	378	2.6 [1.6-4.1]	84.9 [62.5-93.9]	<0.001	
**Country income level**								<0.001
High-income economies	0.9 [0.5-1.5]	[0-3]	10	6746400	15.4 [14.1-16.8]	99.6 [99.5-99.6]	<0.001	

	Prevalence. % (95%CI)	95% Prediction interval	N Studies	N Participants	H (95%CI)	I^2 (95%CI)	P heterogeneity	P difference subtypes
Low-income economies	9.6 [0.4-27.2]	[0-98.1]	4	1674	5.5 [4.1-7.4]	96.7 [94-98.2]	<0.001	
Lower-middle income economies	5 [3.8-6.3]	[0.4-13.4]	30	132980	8.5 [7.9-9.1]	98.6 [98.4-98.8]	<0.001	
Upper-middle-income economies	1.6 [1.3-2.1]	[0.1-4.3]	43	515271	7.4 [6.9-7.9]	98.2 [97.9-98.4]	<0.001	
**Population categories**								<0.001
HBsAg Negative	0.3 [0.2-0.4]	[0-1]	27	567324	5.8 [5.2-6.4]	97 [96.3-97.5]	<0.001	
HBsAg Negative & Anti-HBc Negative	0 [0-0]	[0-0.2]	7	72555	1.4 [1-2.1]	46.7 [0-77.5]	0.081	
HBsAg Negative & Anti-HBc Positive	6.6 [5.7-7.5]	[2.2-12.8]	53	6756446	10.9 [10.5-11.5]	99.2 [99.1-99.2]	<0.001	
**OBI diagnostic method**								<0.001
Classical PCR	1.7 [1.2-2.2]	[0.2-4.4]	27	477140	8 [7.4-8.7]	98.5 [98.2-98.7]	<0.001	
Loop mediated isothermal amplification (LAMP)	35.8 [27.5-44.7]	NA	1	120	NA	NA	1	
Real-time PCR	2.6 [2.2-3.1]	[0.5-5.9]	59	6919065	9.9 [9.4-10.4]	99 [98.9-99.1]	<0.001	
